# Diversity and Horizontal Transfer of Antarctic *Pseudomonas* spp. Plasmids

**DOI:** 10.3390/genes10110850

**Published:** 2019-10-28

**Authors:** Krzysztof Romaniuk, Michal Styczynski, Przemyslaw Decewicz, Oliwia Buraczewska, Witold Uhrynowski, Marco Fondi, Marcin Wolosiewicz, Magdalena Szuplewska, Lukasz Dziewit

**Affiliations:** 1 Department of Bacterial Genetics, Institute of Microbiology, Faculty of Biology, University of Warsaw, Miecznikowa 1, 02-096 Warsaw, Poland; romaniuk@biol.uw.edu.pl (K.R.); decewicz@biol.uw.edu.pl (P.D.); oliwia.buraczewska@gmail.com (O.B.); w.uhrynowski@biol.uw.edu.pl (W.U.); marcin.wolosiewicz@gmail.com (M.W.);; 2 Department of Biology, University of Florence, via Madonna del Piano 6, Sesto Fiorentino, 50019 Florence, Italy; marco.fondi@unifi.it

**Keywords:** Antarctica, biofilm, horizontal gene transfer, plasmid, *Pseudomonas*, transposable element

## Abstract

*Pseudomonas* spp. are widely distributed in various environments around the world. They are also common in the Antarctic regions. To date, almost 200 plasmids of *Pseudomonas* spp. have been sequenced, but only 12 of them were isolated from psychrotolerant strains. In this study, 15 novel plasmids of cold-active *Pseudomonas* spp. originating from the King George Island (Antarctica) were characterized using a combined, structural and functional approach, including thorough genomic analyses, functional analyses of selected genetic modules, and identification of active transposable elements localized within the plasmids and comparative genomics. The analyses performed in this study increased the understanding of the horizontal transfer of plasmids found within *Pseudomonas* populations inhabiting Antarctic soils. It was shown that the majority of the studied plasmids are narrow-host-range replicons, whose transfer across taxonomic boundaries may be limited. Moreover, structural and functional analyses enabled identification and characterization of various accessory genetic modules, including genes encoding major pilin protein (PilA), that enhance biofilm formation, as well as active transposable elements. Furthermore, comparative genomic analyses revealed that the studied plasmids of Antarctic *Pseudomonas* spp. are unique, as they are highly dissimilar to the other known plasmids of *Pseudomonas* spp.

## 1. Introduction

The genus *Pseudomonas* comprises more than 200 species inhabiting a wide range of environments, including soil, water, plants and animals. Pseudomonads tolerate a variety of environmental conditions due to a great metabolic versatility [1]. As they are able to utilize various chemical compounds, including chloroanilines, insecticides or chitin, they find wide application in environmental biotechnology and bioremediation [2,3]. Moreover, some *Pseudomonas* spp. were proven to inhibit the development of phytopathogens and promote plant growth, thus finding application in agriculture [4]. Some *Pseudomonas* spp. are also pathogenic, e.g., *Pseudomonas aeruginosa—*an opportunistic human pathogen that causes severe infections. This pathogen usually exhibits intrinsic resistance to several different types of antibiotics and chemotherapeutics, and thus it is usually difficult to eradicate [5]. 

*Pseudomonas* spp. were also found in permanently cold, polar regions, including Arctic and Antarctic marine waters, glaciers and soil [6,7,8,9]. Studies applying a culture-dependent approach revealed that *Pseudomonas* spp. are (sub)dominant among the Arctic and Antarctic cultivable bacteria [6,10,11].

*Pseudomonas* spp. frequently harbor between one and six plasmids, differing significantly between the strains in terms of size, encoded features, and the host range. The average size of a pseudomonad plasmid is about 113 kb. The smallest identified one (1089 bp) is an unnamed plasmid 2 (GenBank accession number CP033773) of *Pseudomonas aeruginosa* FDAARGOS_532. It encodes only two hypothetical proteins. The largest known plasmid of *Pseudomonas* spp., pMPPla107 (GenBank accession number NZ_CM000959), is harbored by a phytopathogen *Pseudomonas *syringae** pv. lachrymans str. M301315. It is 967,397 bp in size, and constitutes 14% of the host genome [12]. This mega-sized replicon encodes a type IV secretion system and a diverse set of proteins with unknown functions. Although the variability of the *Pseudomonas* plasmids is high, many of them are broad-host-range and self-transmissible. Both these features facilitate their spread via horizontal transfer. This is especially important as *Pseudomonas* plasmids frequently carry phenotypic genetic modules of adaptive value. Many of these replicons are associated with the utilization of toxic compounds and resistance to toxic metals or antibiotics, e.g., pWW0 [13] and NAH7 [14], which encode proteins involved in the degradation of toluene and naphthalene, or pUM505—conferring chromate and mercury resistance [15].

As mentioned above, pseudomonads are frequently isolated from polar environments. However, to our knowledge, to date, only seven plasmids of Antarctic *Pseudomonas* spp. were described. Three of them were found within *Pseudomonas* sp. GLE121. Two of these plasmids are cryptic, while the largest one, pGLE121P3 (39,583 bp), carries a UV-resistance module (a homolog of the *rulAB* operon) and may play a significant role in the adaptation of the host to increased UV radiation in this region [16]. The next two plasmids originated from *Pseudomonas antarctica* PAMC 27494, and the larger one, pP27494_1 (135,475 bp), contains a predicted type III secretion system [17]. The sixth known plasmid, a replicon named KOPRI126573 of *Pseudomonas* sp. MC1, carries genetic modules enabling naphthalene degradation, biofilm formation and increased UV-resistance [18]. Finally, the seventh plasmid of the Antarctic *Pseudomonas* strain, a temperature-sensitive replicon pA3J1, originated from a strain isolated by members of our research group from a soil sample collected at the King George Island. Analysis of this plasmid encouraged us to perform a more detailed study of *Pseudomonas* spp. plasmids from this region, which were described in this work. 

In this study, we have identified and characterized 15 novel plasmids of Antarctic, psychrotolerant *Pseudomonas* spp. This allowed for a unique insight into the phenomenon of the horizontal transfer of plasmids among *Pseudomonas* spp. in Antarctic soils. 

## 2. Materials and Methods 

### 2.1. Bacterial Strains, Plasmids and Culture Conditions

The following laboratory bacterial strains were used: *Achromobacter* sp. LM16R [19], *Agrobacterium tumefaciens* LBA288 [20], *Escherichia coli* BR825 [21], *E. coli* DH5α [22], *Pseudomonas aeruginosa* PAO1161 [23] and *Variovorax paradoxus* EPS [24]. The strains were grown on LB medium at 30 °C (*Achromobacter* sp., *A. tumefaciens* and *V. paradoxus*) or 37 °C (*E. coli* and *P. aeruginosa*). The medium was solidified by the addition of 1.5% (w/v) agar. Where necessary, the medium was supplemented with X-gal, IPTG and antibiotics: kanamycin (50 µg/mL for *A. tumefaciens*, *E. coli* and *V*. *paradoxus* or 500 µg/mL for *Achromobacter* sp. and *P. aeruginosa*) and rifampicin (50 µg/mL). Plasmids used and constructed in this study are listed in Table 1.

### 2.2. DNA Manipulations and Introduction of Plasmid DNA into Bacterial Cells

The DNA of the plasmids was isolated using a GeneMATRIX Plasmid Miniprep DNA Purification Kit (EURx, Gdansk, Poland) or the classical alkaline lysis procedure [30]. Routine DNA manipulations were carried out using standard molecular biology methods [31]. DNA was amplified by PCR using a KAPA HiFi PCR Kit and appropriate primer pairs (Table S1). DNA amplification was performed using a Mastercycler nexus gradient, model number F283971R (Eppendorf, Hamburg, Germany). Each thermocycle started with an initial denaturation at 95 °C for 3 min followed by 30 cycles of denaturation at 98 °C for 20 s, annealing at 56 to 63 °C (depending on the primer pair) for 15 s, extension at 72 °C for 1 min/kb, and finished with a final extension at 72 °C for 1 min/kb. The PCR-amplified DNA fragments were then cloned in the pABW1 or pBBR1MCS-2 vectors.

Plasmids constructed in this study were introduced into *Achromobacter* sp. LM16R, *A. tumefaciens* LBA288, *P. aeruginosa* PAO1161 and *V. paradoxus* EPS by triparental mating [32], and into *E. coli* BR825 by chemical transformation [33,34].

### 2.3. Testing of the Host Range of the Pseudomonas Plasmids

Derivatives of pABW1 carrying replication modules (REP) of *Pseudomonas* plasmids (Table 1) were introduced into *Achromobacter* sp. LM16R, *A. tumefaciens* LBA288, *E. coli* BR825, *P. aeruginosa* PAO1161 and *V. paradoxus* EPS. The ColE1-type replication system of pABW1 is not functional in any of these recipient strains (*E. coli* BR825 carries a mutation within the DNA polymerase I gene that prevents ColE1-type replication). Therefore, maintenance of the shuttle plasmids (pABW1-derivatives) in the tested hosts was dependent on the functions encoded within the cloned, predicted replication modules of the analyzed *Pseudomonas* plasmids. The presence of an introduced plasmid within a host strain was confirmed by DNA electrophoresis.

### 2.4. Testing of Bacterial Adherence to an Artificial Surface

The modified crystal violet staining method was used for testing of bacterial adherence to an artificial surface [35]. Bacteria were cultivated overnight in LB medium supplemented with kanamycin at 37 °C and then diluted to obtain OD_600_ (optical density at the wavelength of 600 nm) = 0.5 (CFU ≈ 4 × 10^8^) in the same medium. In the next step, samples of 200 µL of the cell suspensions (three biological replicates; with twenty technical replicates each) were transferred to the wells of sterile 96-well plates and incubated at the required temperature without shaking. After 24 h, the OD_600_ of the cultures was measured using a Sunrise^TM^ plate reader (with Magellan software version 7.2; Tecan, Männedorf, Switzerland). The medium containing non-adherent cells was then removed, and all wells were rinsed twice with 0.85% NaCl solution and dried at 37 °C for about 15 min. The remaining (adherent) bacterial cells were stained with 0.1% (w/v) crystal violet (200 µL/well) and the plates were incubated at room temperature. After 10 min, the staining agent was removed, and the wells were rinsed twice with saline solution and dried again at 37 °C. The dried stained cell structures (biofilms) were then dissolved by adding 95% ethanol (200 µL/well) and incubating for 10 min. The OD_570_ of the obtained suspension was measured using a Sunrise^TM^ plate reader. Adherence capabilities were assessed by calculating the OD_570_/OD_600_ ratio. 

### 2.5. Statistical Analysis

The statistical significance of the results obtained during testing of bacterial adherence to an artificial surface and comparative genomic analyses of *Pseudomonas* plasmids was determined by the Mann–Whitney U test. The statistical similarity between Antarctic *Pseudomonas* spp. plasmids and Antarctic *Pseudomonas* spp. plasmids in comparison with plasmids of mesophilic *Pseudomonas* spp. was based on all the nucleotide BLAST results with the 1 × 10^−50^ e-value threshold applied. The level of similarity between two plasmids was calculated as the sum of lengths of sequence alignments between them divided by the sum of plasmid lengths. 

### 2.6. Live Cell Confocal Microscopy (Biofilm Analysis)

Scanning Confocal Laser Microscopy (SCLM) was used to image live cells in biofilms [36]. Bacteria were cultivated overnight in LB medium supplemented with kanamycin at 37 °C and then diluted to obtain OD_600_ (optical density at a wavelength 600 nm) = 0.5 (CFU ≈ 4 × 10^8^) in the same medium. In the next step, 3 mL of suspension of each of the bacterial cultures was transferred into sterile, glass-bottom dishes (35 mm diameter, 20 mm glass diameter, no. 1.5 coverslip; MatTek Corporation, Ashland, MA, USA) and incubated without shaking for 24 h at 37 °C. The medium was removed from the dish and the biofilm that had developed on the bottom was washed two times with 10 mM MgSO_4_. Subsequently, the biofilms were stained for 30 min with 3 mL acridine orange (10 µg/mL in 10 mM MgSO_4_; Sigma, Basel, Switzerland) and then rinsed twice with 10 mM MgSO_4_. Confocal microscopy was then performed using a Nikon Eclipse Ti (A1) microscope equipped with a ×60, 1.4 NA oil immersion phase-contrast lens (Nikon Corporation, Tokyo, Japan). An argon laser with a maximum-emission line at 488 nm was used as the excitation source. Horizontal optical thin sections were collected at 0.21-μm intervals from the outer surface of the biofilm to the bottom of the glass plate. These images were captured using NIS-ELEMENTS interactive software version 4.50 (Nikon Corporation) and three-dimensional reconstructions were created.

### 2.7. Testing of the Functionality of Transposable Elements (TEs) Carried within the pA62H2 Plasmid

The broad host range (BHR) entrapment vector pMAT1 was used for testing of the functionality of transposable elements identified in silico within the plasmid pA62H2. This entrapment vector was introduced into the rifampicin-resistant AH62LR strain by triparental mating, as described previously [32]. The pMAT1 contains, as the selection cassette, the *sacB* gene originating from *Bacillus subtilis*. The product of this gene (ectoenzyme levanosacharase), in the presence of sucrose, synthesizes levans which are lethal for Gram-negative bacteria [37]. Therefore, only the clones carrying an entrapment plasmid in which the *sacB* gene is inactivated (e.g., by the insertion of a TE), can grow on the selective medium with sucrose (selection of Suc^r^ mutants). Overnight culture of AH62LR (containing pMAT1) was plated on the selective medium with sucrose, as well as non-selective LB medium (which enabled the determination of the frequency of mutations within the *sacB* gene). The plasmid pattern of 100 Suc^r^ clones was analyzed by DNA electrophoresis. The selected plasmids, containing potential inserts, were introduced using the chemical transformation method [33] into the *E. coli* DH5α. The insertion sites of the elements were localized by PCR, with the pMAT1 insertion derivatives as template DNA, and the previously described sets of cassette-specific primers (Table S1) [28]. The selected primers were used for sequencing of the terminal parts of the identified TEs.

### 2.8. DNA Sequencing

DNA sequencing was performed using an Illumina MiSeq instrument in paired-end mode using a v3 chemistry kit. The obtained sequence reads were filtered for quality using cutAdapt v1.9.0 (trimming bases on 3’ ends with quality lower than 20 and removing reads shorter than 100 bp) and assembled using Newbler v3.0 software (Roche, Basel, Switzerland). Final gap closure and TEs sequencing were performed by capillary sequencing of PCR products using an ABI3730xl DNA Analyser (Applied Biosystems, Waltham, MA, USA) applying a primer walking technique. The summary of the sequencing of particular *Pseudomonas* plasmids was presented in Table S2.

### 2.9. Bioinformatics

Plasmid genomes were manually annotated using the Artemis software [38]. Similarity searches were performed using the Basic Local Alignment Search Tool (BLAST) [39], Pfam tool [40] and Conserved Domains Database [41], provided by the National Center for Biotechnology Information [42] (NCBI; http://blast.ncbi.nlm.nih.gov/Blast.cgi). The detection of RNA sequences was performed using tRNAscan-SE programs [43]. Helix-turn-helix motifs were identified using the helix-turn-helix DNA-binding motif prediction tool (https://npsa-prabi.ibcp.fr/cgi-bin/npsa_automat.pl?page=/NPSA/npsa_hth.html) [44]. EC numbers were assigned using the KEGG database [45] and UniProt Knowledgebase (UniProtKB) [46]. Insertion sequences were analyzed using the ISfinder database [47].

The 16S rDNA-based phylogenetic analysis was performed using the MEGA6 tool [48]. The unrooted tree was constructed using the maximum-likelihood algorithm (with the Tamura-Nei model), and statistical support for internal nodes was determined by 1000 bootstrap replicates. For the UPGMA (unweighted pair group method with arithmetic mean) analysis, the results of the determination of metal minimum inhibitory concentrations (performed in our previous study [6]) were used. For each metal, the data were normalized by division of the result for each strain by the highest obtained MIC result for the particular metal. The UPGMA dendrogram was created using the MEGA6 tool [48]. 

Genomic comparison of 208 *Pseudomonas* plasmids was performed with the Circoletto tool [49] with the e-value threshold of 1 × 10^−50^. During the analysis, four thresholds of sequence identity were shown: 71%–80% (represented as blue ribbons), 81%–90% (green), 91%–95% (orange), and 96%–100% (red). To increase the visibility, all plasmids identified and characterized in this study were (proportionally to their size) enlarged five times compared to the other plasmids. The construction of similarity networks was based on all-against-all BLASTp comparisons of the predicted proteomes of the analyzed plasmids. The following thresholds were used during the BLASTp searches: e-value 1 × 10^−10^ (to avoid losing small, i.e., < 100 aa, proteins from the analysis), query coverage of HSP and sequence identity of at least 90%. Within the obtained networks, each node represents a single plasmid and each edge corresponds to a common reciprocated similarity of at least one protein encoded by two connected plasmids. The size of the node corresponds to the relative size of the plasmid. The thickness of the edge reflects the number of common proteins between two plasmids being compared. The network was created using self-written Python scripts [50] and visualized in Gephi using ForceAtlas 2 layout [51].

### 2.10. Nucleotide Sequence Accession Numbers

The nucleotide sequences of the *Pseudomonas* spp. plasmids identified in this study were deposited in the GenBank (NCBI) database with the accession numbers MK376337–MK376351.

## 3. Results and Discussion 

### 3.1. Distribution of Plasmids in Antarctic Pseudomonas spp.—Evidences for the Plasmid Horizontal Transfer

In our previous study, 57 bacterial strains of the genus *Pseudomonas* were isolated from soil samples collected at the King George Island, Antarctica. Pseudomonads were identified in soils originating from the Jardine Peak and the Arctowski Polish Polar Station. Strains were thoroughly characterized, and the majority of them revealed to be metalotolerants, as they were resistant to As(III)—28 strains; As(V)—53; Co(II)—1; Cu(II)—44; Ni(II)—38; Zn(II)—13. It was also revealed that some of the strains exhibited joint resistance to two (11 strains), three (20), four (7) or five (12) metals [6]. 

In this study, plasmid screening (applying alkaline lysis) of all the above-mentioned *Pseudomonas* strains was performed. The alkaline lysis allows isolation of plasmids usually of up to 150–200 kb. Performed analysis showed that 18 (31.6%) strains harbor between one and three plasmids. In total, 26 plasmids were detected. Additionally, in the course of our previous studies, one more plasmid—pA3J1 of *Pseudomonas* sp. ANT_J3 (from the same collection of Antarctic bacterial strains)—was detected and characterized [26]. All identified plasmids were isolated using a GeneMATRIX Plasmid Miniprep DNA Purification Kit (EURx) or the classical alkaline lysis, and then they were subjected to DNA sequencing. After obtaining the full genomic sequences of all the plasmids (i.e., closing circular DNAs), it was shown that only 15 replicons are unique (Table 2). This gives us an opportunity to track the possible plasmid horizontal transfer in the studied *Pseudomonas* population. 

**Figure 1:**
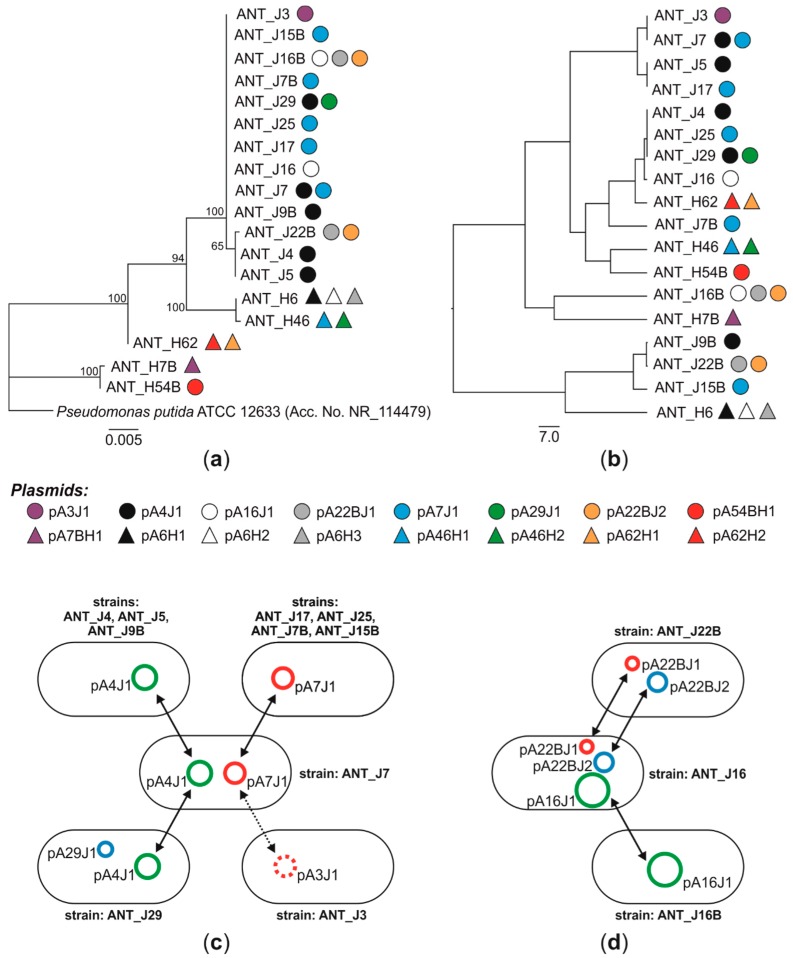
Plasmid occurrence and possible directions of the horizontal transfer of plasmids between the selected *Pseudomonas* strains isolated from the Antarctic soil. (**a**) The phylogenetic tree of plasmid-carrying Antarctic *Pseudomonas* spp. constructed based on 16S rDNA sequences. The unrooted tree was constructed using the maximum-likelihood algorithm (with the Tamura-Nei model), and statistical support for internal nodes was determined by 1000 bootstrap replicates. Values of >50% are shown. *Pseduomonas putida* ATCC 12633 was used as an out-group. (**b**) The UPGMA dendrogram was constructed based on results of analysis of heavy-metal resistance of Antarctic *Pseudomonas* spp. Circles and triangles on both trees indicate particular plasmids (identified in this study, plus pA3J1, characterized previously [26]). (**c**,**d**) The schemes showing the distribution of: (**c**) pA3J1, pA4J1, pA7J1 and pA29J1 plasmids, and (**d**) pA16J1, pA22BJ1 and pA22BJ2 plasmids. Plasmids with the same names but occurring in various strains are identical (100% of nucleotide sequence identity). Arrows indicate the possible directions of horizontal transfer of the analyzed plasmids. A dotted line indicates (potential) horizontal transfer of plasmid along with its possible point mutations.

It was revealed that the most widespread plasmids were pA4J1 (10,609 bp) and pA7J1 (9794 bp). Each of these plasmids was detected in five bacterial strains. We identified a strain (ANT_J7) harboring both plasmids, as well as hosts carrying exclusively pA4J1 (strains: ANT_J4, ANT_J5 and ANT_J9B) or pA7J1 (ANT_J17, ANT_J25, ANT_J7B and ANT_J15B) (Figure 1c). It was also shown that the pA4J1 plasmid may co-occur with another plasmid, pA29J1 (3214 bp) in the same host, i.e., the ANT_J29 strain. Interestingly, pA3J1—the only plasmid detected within the ANT_J3 strain in the course of our previous studies [26]—differs from pA7J1 only by 6 bp (Figure 1c). This finding may indicate the possibility of a horizontal transfer of the pA7J1 plasmid and subsequent accumulation of mutations. Such phenomena were previously observed for clinically relevant plasmids conferring antibiotic resistance, e.g., pP10159-3 of *Citrobacter freundii* P10159 and pHS062105-3 of *Klebsiella pneumoniae* HS062105 [52].

Possible horizontal transfer of plasmids can also be observed for the strains ANT_J16 (harbors plasmids pA16J1, pA22BJ1, and pA22BJ2), ANT_J22B (pA22BJ1 and pA22BJ2) and ANT_J16B (pA16J1) (Figure 1d). Interestingly, the plasmid pA22BJ2 (7521 bp) was revealed to be closely related with pA4J1. It carries an insertion of 3088-bp DNA fragment (coordinates 1982–5069) encoding: (i) a predicted recombinase (pA4J1_p04), (ii) an IscR family transcriptional regulator (pA4J1_p05) and (iii) a NAD(P)/FAD-dependent oxidoreductase (pA4J1_p06). Acquisition of various genomic islets by small plasmids was shown previously for other replicons, e.g., the plasmids pMARC1 and pMOS7 of *Paracoccus marcusii* [53]). 

The abovementioned hypothesis assumes that the distribution of the same plasmids in different configurations amongst Antarctic *Pseudomonas* strains is a consequence of their horizontal transfer. This is even more feasible as their host strains in most cases differ in terms of the sequences of the 16S rRNA marker genes and/or phenotypic characteristics (Figure 1) [6]. This strongly suggests that these are divergent *Pseudomonas* strains that acquired plasmids and not progeny of a common ancestor harboring a full set of plasmids, that simply lost some of them in the course of evolution (and this resulted in separate evolutionary lines).

In the literature, there are works describing horizontal transfer of genes in an Antarctic environment [54,55,56,57]. However, these are mostly genomic analyses showing the presence of identical DNA fragments in genomes of various Antarctic bacteria. It was also shown that such transfers may have an influence on adaptation and survival chances of the host cells under harsh environmental conditions, e.g., the horizontal transfer of the plasmid-carried *ndo* gene, encoding naphthalene dioxygenase (NDO) among Antarctic *Pseudomonas* strains [58]. However, to our best knowledge, this is the first work providing evidence for in situ horizontal transfer of entire plasmids between bacteria co-residing in the Antarctic soil.

### 3.2. Diversity of Plasmids of Antarctic Pseudomonas spp.

#### 3.2.1. Replication Modules

**Figure 2:**
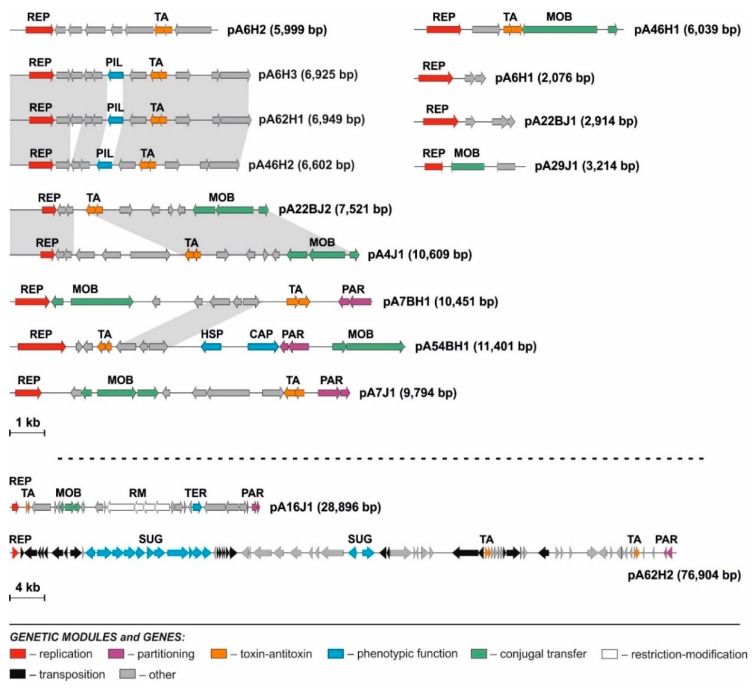
Linear maps showing the genetic structure and organization of the circular plasmids of the Antarctic *Pseudomonas* spp. Arrows indicate genes and their transcriptional orientation. The predicted genes/genetic modules: CAP—capsule polysaccharide export system; HSP—heat shock protein; MOB—mobilization to conjugal transfer; PAR—partitioning; PIL—pilus assembly; REP—replication; RM—restriction–modification system; SUG—type I polysaccharide secretion; TA—toxin–antitoxin; TE—transposable element; TER—tellurium resistance are indicated.

An in-depth bioinformatic analysis of the sequenced *Pseudomonas* plasmids allowed identification of their replication modules, REP (Figure 2, Tables S3 and S4). REP are basic genetic cassettes of plasmids that enable their autonomous replication in host cells, commonly used for classification of these replicons [59,60,61]. 

Comparisons with the reference representatives of the previously distinguished incompatibility groups enabled classification of seven replication modules of the analyzed plasmids (i.e., pA6H3, pA7J1, pA16J1, pA46H2, pA54BH1, pA62H1 and pA62H2) into the IncP incompatibility group. Among them, only three REP modules (of the plasmids pA6H3, pA46H2 and pA62H1) were classified into a specific subgroup, namely IncP-7. The replication systems of the remaining plasmids showed limited (less than 50% of aa identity of the replicase protein) or did not reveal any significant similarity to the reference replication systems, which indisposed their classification into defined Inc groups. 

To identify putative origins of replication (*oriV*s) within the REP modules of Antarctic *Pseudomonas* plasmids, DNA regions upstream and downstream of the predicted *rep* genes were searched for AT-rich sequences (where the replication process might be initiated) and iteron-like repeats (i.e., invert or direct repeats where Rep proteins might bind) [62,63]. This analysis allowed the identification of the predicted *oriV*s in all the analyzed plasmids, suggesting that these are iteron-containing replicons (Table S4).

The host ranges of the replication modules (of the identified *Pseudomonas* spp. plasmids) were tested. As the hosts *E. coli* BR825, *P. aeruginosa* PAO1161 (both belonging to *Gammaproteobacteria*), *A. tumefaciens* LBA288 (*Alphaproteobacteria*), *Achromobacter* sp. LM16 and *Variovorax paradoxus* EPS (*Betaproteobacteria*) were selected. The replication system of pA6H1 exhibited the broadest host range, since it was functional in *E. coli* BR825, *P. aeruginosa* PAO1161 and *A. tumefaciens* LBA288. The REP modules of pA54BH1, pA7BH1 and pA62H2 were functional in *Pseudomonas* spp. and *A. tumefaciens* LBA288. The remaining plasmids had narrow host ranges limited to bacteria of the genus *Pseudomonas*. All of the tested REP modules were not able to replicate in the tested members of the *Betaproteobacteria* class. The analysis revealed that 73% of the analyzed plasmids had narrow host ranges, limited to *Pseudomonas* spp. Interestingly, the majority of plasmids of Arctic and Antarctic bacteria examined so far also had narrow host ranges [26,64,65,66], suggesting that this phenomenon is common in permanently cold regions.

Horizontal transfer can be perceived as a parasitic trait of the mobile genetic elements because of its overall costs to the host cell, but, on the other hand, also as a trait benefiting host populations through the sharing of a common gene pool [67]. It was confirmed by the large scale meta-analysis that phylogenetic distance is the greatest barrier to gene sharing [68]. This limited transfer across phylogenetic classes could result from various circumstances, e.g., restriction (due to the presence of endogenous restriction-modification systems) or non-active (or incompatible) replication systems (i.e., narrow host range) [69]. An open question remains, what is the ecological role of narrow versus broad host range, and what is the influence of various environmental conditions on the prevalence of each strategy.

It was suggested that there are different benefits of narrow and broad host ranges of plasmids, and that they depend on the environmental conditions, i.e., under conditions that do not select for altruism (sharing goods), bacteria promoting plasmid transfer may be outcompeted by hosts with lower transfer rate [67]. Therefore, narrow host range seems to be beneficial when the plasmids confer competitive advantages to their hosts, as this gives an ecological supremacy of a given population (e.g., under extreme environmental conditions), while a broad host range is beneficial when the plasmids encode a public goods (cooperative traits) and the environmental conditions are favorable [70,71]. 

In the light of this theory, abovementioned hypothesis that the prevalence of narrow host range plasmids is a common phenomenon in permanently cold regions (and especially Arctic and Antarctica) seems to be justified. In these regions, due to limitation of substrates, deficiency of easily accessible water and detrimental physical conditions (i.e., permanent cold) sharing goods between bacteria is not profitable. Although, this theory seems to be reasonable, for its reliable confirmation a high-throughput screening of host ranges of plasmids from Arctic and Antarctic regions is still needed.

#### 3.2.2. Mobilization to Conjugal Transfer Modules

Nine of the identified Antarctic *Pseudomonas* spp. plasmids carry predicted MOB (mobilization to conjugal transfer) modules (Figure 2). The bioinformatic analyses of relaxases (MobAs) lead to the identification of conserved motives characteristic for particular families of these proteins [72,73]. Six identified MobAs can be assigned to the MOB_Q_ family, including pA4J1, pA7J1, pA16J1, pA22BJ2, pA7BH1 and pA46H1. The MobAs of the plasmids pA29J1 and pA54BH1 were classified into MOB_V_ and MOB_HEN_ families, respectively. For seven plasmids, predicted *oriTs* were identified based on the homology to the previously distinguished *oriTs* (Table S5). In the case of pA54BH1, it was impossible to determine the predicted *oriT*, since any conserved DNA region showing homology to known *oriTs* was found.

#### 3.2.3. Stabilization Modules

Within the analyzed plasmids, two types of stabilization systems were identified (Figure 2). The first type, partitioning (PAR) module, is responsible for equal distribution of plasmid copies to daughter cells [74]. Such a system was found within five of the analyzed replicons. All of these modules were composed of two predicted genes, encoding ParA-like ATPase and ParB-like protein, respectively. PAR modules of pA7J1 and pA16J1 showed a high level of identity of the amino acid sequences of ParA (95%) and ParB (80%) proteins. The PAR modules of pA7BH1, pA54BH1 and pA62H2 were more diverse. For all of the PAR modules, the putative centromere-like (*parS*) sites—DNA regions containing direct (4- to 6-bp long) repeats—were detected (Table S6).

The second group of stabilization modules found within the *Pseudomonas* spp. plasmids were toxin–antitoxin (TA) systems, that enable post-segregational elimination of plasmid-less cells from the bacterial population [75]. Thirteen TA systems were identified within the analyzed replicons. Eleven of them (found within the plasmids: pA4J1, pA7J1, pA7BHJ1, pA22BJ2, pA6H2, pA6H3, pA16J1, pA46H1, pA46H2, pA62H1 and pA62H2) represented the RelBE family, which is one of the largest TA families encoding toxins acting as side-specific ribonucleases [76]. 

#### 3.2.4. Predicted Phenotypic Modules

Within the analyzed plasmids, several predicted phenotypic modules were found (Figure 2, Table 2). For example, the putative tellurium resistance module (TER; *pA16J1_p21*) was found within pA16J1. The tellurium toxicity is related to the generation of reactive oxygen species disturbing the thiol:redox buffering system. As has previously been shown, tellurium-resistant bacteria are common in Antarctic regions [77,78,79,80]. The TER module found within pA16J1 is formed by a single gene showing homology to *terC* genes previously identified in *Arthrobacter* spp. plasmids isolated from the same soil sample [81]. It was shown before that the single *terC* gene is not sufficient for conferring tellurium resistance [81]. 

In addition, two putative phenotypic modules were found in the pA54BH1 plasmid, situated next to each other. The *pA16J1_p09* is a gene encoding a putative heat-shock protein (HSP), that is possibly involved in protection against temperature changes or switching on/off the stationary phase of growth [82]. Another gene, *pA16J1_p10*, encodes a putative capsule polysaccharide export protein (CAP) [83].

The largest phenotypic module was found within the pA62H2 plasmid and it comprised a type I polysaccharide secretion system (SUG). It is composed of 11 genes (*pA62H2_p11*-*p21*), encoding putative: (i) glycosyl transferase of family 1 (EC 2.4.1.11; *pA62H2_p12*), (ii) ATPase (*pA62H2_p12*), (iii) membrane fusion protein of the HlyD family (*pA62H2_p13*), (iv) TolC-like outer-membrane protein (*pA62H2_p14*), (v) GDP-mannose 4,6-dehydratase (EC 4.2.1.47; *pA62H2_p15*), (vi) lipopolysaccharide permease (*pA62H2_p16*), (vii) ATP-binding transporter (*pA62H2_p17*), (viii) D-inositol-3-phosphate glycosyltransferase (*pA62H2_p18*), (ix) a hypothetical protein (*pA62H2_p19*), (x) methyltransferase (*pA62H2_p20*) and (xi) acyltransferase (*pA62H2_p21*). Additionally, within the same plasmid, two other genes potentially belonging to the SUG module were also identified. These were *pA62H2_37* and *pA62H2_38* encoding predicted GDP-6-deoxy-D-lyxo-4-hexulose reductase (EC 1.1.1.281) and mannose-1-phosphate guanyltransferase (EC 2.7.7.13), respectively. 

Among the identified phenotypic modules, genes encoding major pilin protein (PilA) were found. PilA proteins are responsible for the initiation of surface attachment, which is the first stage of biofilm formation [84] and the pilin mutants were shown to be defective in performing this process [85]. The PIL modules were found within pA6H3, pA46H2 and pA62H1. Interestingly, these three replicons are nearly identical, differing within the DNA regions comprising the PIL modules. The comparison of the PilA proteins encoded by these plasmids showed that they are homologous, but the differences between them are significant. PilA of pA6H3 showed only 44% identity with its homolog of pA46H2 and 70% with PilA of pA62H1, while the comparative analysis of the proteins from pA46H2 and pA62H1 showed only 50% identity. These three PIL modules were subjected to further functional analyses.

#### 3.2.5. Functional Analysis of the PIL Modules—Biofilm Synthesis Testing

**Figure 3:**
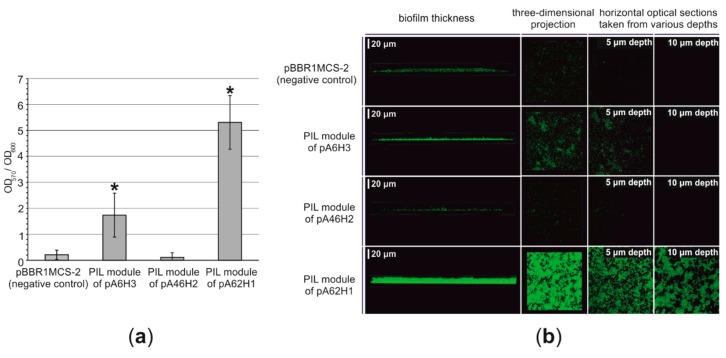
(**a**) Attachment of *E. coli* DH5α harboring the plasmids pBBR1MCS-2, pBBR1-Pil6 (with PIL module of pA6H3), pBBR1-Pil46 (with the PIL module of pA46H2) and pBBR1-Pil62 (with the PIL module of pA62H1) to the polystyrene surface of microtitration plates, assessed by crystal violet staining. Error bars represent standard deviations. *—mean statistical significance *p* < 0.0001, compared to the adherence of *E. coli* DH5α harboring plasmids pBBR1MCS-2 (negative control). (**b**) Biofilm structure of *E. coli* DH5α harboring plasmids pBBR1MCS-2, pBBR1-Pil6 (with the PIL module of pA6H3), pBBR1-Pil46 (with the PIL module of pA46H2) and pBBR1-Pil62 (with the PIL module of pA62H1) after 24 h of cultivation, visualized by scanning confocal laser microscopy. Scale bars for biofilm thickness are presented. Horizontal optical sections were recorded at depths of 5 and 10 µm from the bottom of the dish.

The PIL modules of pA6H3, pA46H2 and pA62H1 were cloned into pBBR1MCS-2 vector, resulting in pBBR1-Pil6, pBBR1-Pil46, pBBR1-Pil62 plasmids. To investigate the functionality of each PIL module, *E. coli* DH5α derivatives carrying plasmids pBBR1-Pil6, pBBR1-Pil46, pBBR1-Pil62 or “empty” pBBR1MCS-2 vector (as a control) were used for the bacterial adherence test (Figure 3a). The modified crystal violet staining method was used [35].

The obtained results indicated that the PIL module of pA62H1 enhances the attachment of bacterial cells (Figure 3a). The attachment ability of bacteria is significantly (*p* < 0.0001) increased in the presence of pBBR1-Pil62. Analogically, the introduction of the PIL module of pA6H3 to bacterial cells also resulted in their stronger adherence to the artificial (polystyrene) surface (*p* < 0.0001; however, this is weaker than in the case of the previous module) (Figure 3b; Figure S1). In contrast, no enhancement of the surface adhesion was observed in cells to which the plasmid pBBR1-Pil46, carrying the PIL module of pA46H2 was introduced (Figure 3b; Figure S1). This finding indicates that the PIL modules of plasmids pA6H3 and pA62H1 are functional, while that of pA46H2 may be inactive in the tested host or otherwise impaired.

As stated before, the crystal violet staining assay should only be considered a preliminary test of the adherence abilities of bacterial strains [86]. Therefore, an attempt was made to visualize the biofilm structures using scanning confocal laser microscopy (SCLM). In the case of strains harboring plasmids pBBR1-Pil62 and pBBR1-Pil6, a significantly thicker and denser biofilm was observed compared to the control strain (Figure 3b). This confirmed that the PIL modules of the plasmids pA6H3 and pA62H1 enhance biofilm formation. 

Plasmid-enhanced adherence and biofilm formation abilities may be highly advantageous in permanently cold environments. It was proposed that such abilities may increase bacterial survival in permafrost by enhancing attachment in the proximity of liquid water (a limited resource in cold regions) and protection of bacterial cells against osmotic shock, desiccation and UV radiation [87,88,89,90]. It is also important to mention that biofilms may also facilitate horizontal gene transfer in Antarctic regions due to the presence of extracellular DNA and enhanced DNA acquisition in these structures, as observed in previous studies [91].

#### 3.2.6. Transposable Elements Present in the *Pseudomonas* Plasmids

Transposable elements were identified exclusively in the pA62H2 plasmid. In silico analysis of this replicon revealed four complete transposable elements, i.e., (i) transposon Tn*5501* of the Tn*3* family and (ii) three novel insertion sequences IS*Psp13*, IS*Psp14* and IS*Psp15* of the IS*4*, IS*3*, and IS*256* families, respectively (Table 3). The nucleotide sequences of the identified ISs have been deposited in the ISfinder database. Moreover, within the pA62H2 plasmid, four partial insertion sequences (belonging to IS*66*, IS*3* and IS*110* families) were identified. 

A non-composite transposon Tn*5501* of pA62H2 shows 98% nucleotide sequence identity with a TE found within the pPGH1 plasmid of *Pseudomonas putida* H [92]. However, this specific TE also occurs in many other plasmids of *Pseudomonas* spp., including pPC9 of *P. putida* HB3267 [93] and pND6-1 of *P. putida* ND6 [94]. Tn*5501* has non-identical 38 bp-long IR sequences and contains six genes. Among them, two genes create a predicted *tad-ata*-like toxin–antitoxin system (of *relBE* family), homologous to the module identified in the plasmid pAMI2 of *Paracoccus aminophilus* JCM 7686 [95]. Therefore, the toxin–antitoxin system of Tn*5501* is an interesting example of a portable stabilization module. 

Three insertion sequences identified in pA62H2 are typical ISs, encoding transposases exclusively. In the case of IS*Psp13* and IS*Psp15*, one gene for transposase was annotated, while IS*Psp14* contains two genes. One of these genes has a conserved frameshift motif 5’-AAAAAAT-3’ (position: 265–271), which is likely to promote the formation of a fusion protein (predicted, active transposase) as a result of a programmed translational frameshifting. This is the example of negative regulation of the transposition, which is typical for ISs belonging to the IS*3* family [96]. 

To verify the transposition activity of the TEs identified in silico within the pA62H2 plasmid, an entrapment vector pMAT1 was introduced into a rifampicin-resistant AH62LR strain. The Km^r^ Suc^r^ clones were selected on LB medium supplemented with sucrose with a frequency of 1.3 × 10^−5^. The plasmid pattern of 100 such clones was analyzed. It was revealed that only Tn*5501* was able to transpose into the selective cassette of pMAT1. The frequency of Tn*5501* transposition was 2.6 × 10^−6^. Transposition of this element into the *sacB* gene of pMAT1 resulted in formation of a 5 bp duplication of target sequences (DRs) (5′-CGATT-3’ or 5′-AACTA-3’). This result may suggest that the ISs found within pA62H2 are either inactive, or there are no specific target sequences for these ISs within the selection cassette of pMAT1. 

### 3.3. Comparative Analysis of the Pseudomonas spp. Plasmids

**Figure 4:**
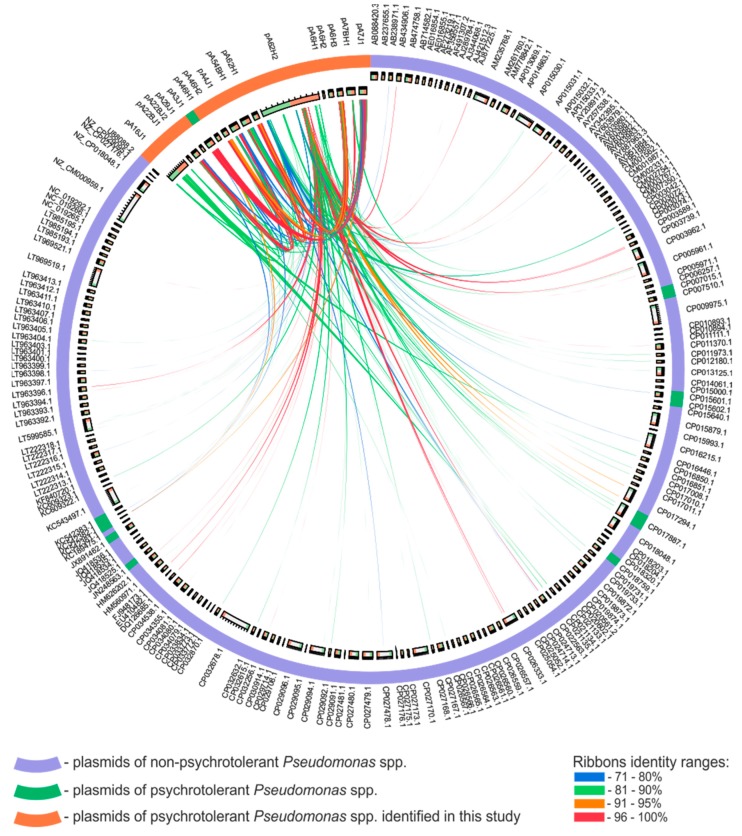
Nucleotide sequence-based comparison of the plasmids identified in this study (plus pA3J1, that was identified previously and originates from the same environment) with 192 *Pseudomonas* spp. plasmids retrieved from the GenBank (NCBI) database. Whole-plasmid-genome similarity analysis was performed using Circoletto, with 1 × 10^−50^ as the threshold. Plasmid sequences are represented as ideograms on the innermost ring. The size of the ideograms is proportional to the size of the plasmids; however, the plasmids identified in this study and pA3J1 were enlarged five times for a better visibility. The start and end positions of each plasmid genome are represented as green and orange blocks within the ideograms. The outer rings indicate the psychrotolerance of the host strain and name or accession number of the plasmid, respectively. The ribbon colors reflect the percentage identity of particular genomic regions.

*Pseudomonas* spp. plasmids identified in this study, along with the previously described pA3J1 [26], were compared with 195 other *Pseudomonas* plasmids retrieved from the GenBank database. Comparison of their nucleotide sequences revealed a limited number of identical/similar regions (Figure 4). Only five highly similar DNA regions (at least 1000-bp long and showing at least 90% of reciprocal identity) were found. The first one is the 2194 bp-long region containing the MOB module of pA7J1 and pA3J1 that is homologous (93% nt identity) to an appropriate module of pGLE121P1 (GenBank acc. no. KC542381) isolated from the Antarctic glacier [16]. Analogically, the second region of homology (1030 bp-long fragment of pA29J1) also contains a gene encoding MobA protein. This DNA region shows 93% identity with the MOB module of the pPFS plasmid of *Pseudomonas fragi* A22 (GenBank acc. no. KX353854). The host of this small (2547 bp), cryptic plasmid was isolated from Arctic soil. The third homologous DNA fragment (1952 bp-long), present within the plasmids pA7BH1 and pA54BH1, carries three genes (encoding a putative resolvase and two hypothetical proteins). It shows 99% of sequence identity with a part of the pZM1P1 plasmid (GenBank acc. no. KJ940992) identified within a *Pseudomonas* strain isolated from a post-flotation tailings pond, Zelazny Most, in Poland [97]. The fourth homologous DNA segment (4393 bp-long), containing a part of restriction–modification module of pA16J1 is homologous to the unnamed plasmid 2 (GenBank acc. no. CP021134) of *Pseudomonas fragi* NMC25 isolated from meat in China. The last, longest (5632 bp-long) homologous DNA fragment, is a transposon of the Tn*3* family found within pA62H2 and other *Pseudomonas* plasmids, including pPC9 (GenBank acc. no. CP003739), pND6-1 (GenBank acc. no. AY208917), p1160-VIM (GenBank acc. no. MF144194) and many others.

**Figure 5:**
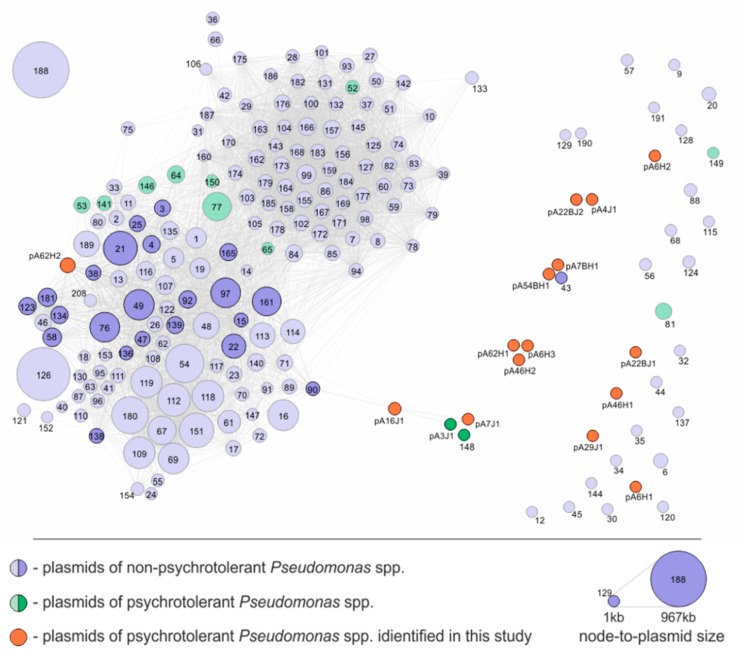
Protein-based similarity network of *Pseudomonas* spp. plasmids. The network was constructed based on the comparison of the predicted proteins encoded by each plasmid compared all-against-all using BLASTp with e-value of 1 × 10^−10^, query coverage of HSP and sequence identity of at least 90% thresholds. Each node (circle) represents a single plasmid. Its size corresponds to the size of the plasmid and its color reflects psychrotolerance characteristics of the host strain. The color intensity of the node reflects the presence (intense color) or absence (bleached color) of at least one protein homologous to a protein encoded in at least one plasmid identified in this study. The number of homologous proteins encoded in two compared plasmids is reflected by the thickness of the edge (link) connecting each pair of nodes. For the clarity of the network, only the names of plasmids identified in this study (plus pA3J1) were shown, while the other plasmids are represented by numbers that correspond with the list of plasmids presented in the Supplementary Table S7.

The comparative genomic analysis of *Pseudomonas* plasmids was supplemented by constructing a protein-based similarity network (Figure 5). The edges between the nodes (i.e., plasmids) were set if at least one protein encoded within a particular plasmid was homologous (cutoff values: 90% query coverage and 90% identity). This approach resulted in the creation of a large cluster, composed of 172 elements, while the other analyzed replicons were singletons or formed small clusters (Figure 5). The majority (80%) of the plasmids characterized in this study was localized separately from the abovementioned large cluster and they mostly formed single (not linked with any other) nodes, which exemplify their uniqueness. Only three plasmids, pA7J1, pA16J1 and pA62H2 of the analyzed Antarctic *Pseudomonas* strains were linked with the main cluster of the created network (Figure 5). However, amongst them, only pA62H2 was localized within the main clique, while pA7J1 and pA16J1, showing limited similarities with other plasmids of the main cluster, were placed on its borders (Figure 5). 

We speculated that the separation of Antarctic plasmids characterized in this study from other replicons of *Pseudomonas* spp. included in the similarity network may be a consequence of the geographical isolation of their hosts. To test that, all other plasmids of psychrotolerant *Pseudomonas* spp. were tested. It was found that seven (out of 12) such replicons originated from Antarctic bacteria, and all these strains were collected at the King George Island. Interestingly, four plasmids (i.e., pP27494_1, pP27494_2, KOPRI126573 and pGLE121P3; numbers 64, 65, 141 and 150 in Figure 5, respectively) were localized within the main cluster of the similarity network (Figure 5). This suggests that geographical isolation of the hosts is probably not the only factor, determining uniqueness of Antarctic plasmids characterized in this study. Analysis of metadata for these four plasmids showed that they originated from bacterial hosts isolated from freshwater (pP27494_1 and pP27494_2), waste water treatment facility (KOPRI126573) and surface ice of Ecology Glacier (pGLE121P3). Therefore, any of these plasmids was isolated from soil bacterium, while all hosts of replicons identified in this study originated from soil samples. It was also shown, that hosts of the remaining four plasmids (i.e., pCC1557, pLIB119, unnamed1 and pD2RT; numbers 52, 53, 77 and 146 in Figure 5, respectively) of psychrotolerant, non-Antarctic bacteria, that were localized within the main cluster of the network, originated from environments other than soil, i.e., snow, marine sediments, glacial stream and sea water, respectively. This observation suggests that possibly summarized effect of the geographical isolation of the plasmid-carrying bacteria and the type of environment they originate may be a reason for a dissimilarity of the plasmids analyzed in this study to other *Pseudomonas* plasmids. However, for a proper testing of this hypothesis, further analyses applying larger sets of data are required.

## 4. Conclusions

In this study, 15 novel plasmids of Antarctic *Pseudomonas* spp. were identified, sequenced and thoroughly analyzed. This tripled the number of known plasmids of cold-active pseudomonads. The analyses performed in this study allowed the horizontal transfer of plasmids occurring within *Pseudomonas* populations inhabiting Antarctic soils to be tracked. It was revealed that identical plasmids can be found in Antarctic *Pseudomonas* strains, characterized by different plasmid patterns and physiological characteristics.

In this study, all the identified plasmids were thoroughly characterized, and genetic modules constituting both their conserved backbones and accessory load were identified. The analysis of the replication modules revealed that the majority of the analyzed plasmids are narrow-host-range replicons. This may limit their transfer to *Pseudomonas* spp. only. Similar observations for other plasmids harbored by Arctic and Antarctic isolates suggest that this phenomenon is common in permanently cold regions. 

Among the analyzed *Pseudomonas* plasmids, three replicons—pA6H3, pA46H2 and pA62H1—carried *pilA*-like genes, which may enhance biofilm formation. Functional analyses of these genetic modules revealed that those of pA62H1 and pA6H3 significantly increase biofilm formation abilities of the host strain, while the PIL module of pA46H2 is inactive in a tested host.

Comparative genomic analyses revealed that Antarctic *Pseudomonas* plasmids are significantly (*p* < 0.0001) more similar to one another than to other, previously identified plasmids of mesophilic bacteria belonging to this genus (Figure S2). This may be surprising, taking into account that in the databases, there are nearly 200 plasmids of *Pseudomonas* spp. On the other hand, it is promising in the light of future analyses of Arctic and Antarctic plasmidomes, which may reveal novel replicons with unique and interesting genetic modules and may shed new light on the phenomenon of horizontal gene transfer occurring in pristine, polar environments. 

## Figures and Tables

**Table 1:** Plasmids used and constructed in this study.

Plasmid Name	Characteristics ^1^	Reference
pABW1	Km^r^; *ori* pMB1; Mob^+^; *oriT* RK2; *lacZ*α; MCS	[25]
pABW1-R	pABW1 carrying the REP module of pA3J1 (SphI/EcoRI restriction fragment)	[26]
pABW1-REP4J1	pABW1 carrying the REP module of pA4J1 (XbaI restriction fragment)	This work
pABW1-REP6H1	pABW1 carrying the REP module of pA6H1 (PCR- amplified with the primers LREP6H1 and RREP6H1)	This work
pABW1-REP6H2	pABW1 carrying the REP module of pA6H2 (PCR- amplified with primers LREP6H2 and RREP6H2)	This work
pABW1-REP6H3	pABW1 carrying the REP module of pA6H3 (PCR- amplified with the primers LREP6H3 and RREP6H3)	This work
pABW1-REP7J1	pABW1 carrying the REP module of pA7J1 (PCR- amplified with the primers LREP7J1 and RREP7J1)	This work
pABW1-REP16J1	pABW1 carrying the REP module of pA16J1 (PCR- amplified with the primers LREP16J1 and RREP16J1)	This work
pABW1-REP22BJ1	pABW1 carrying the REP module of pA22BJ1 (PCR- amplified with the primers LREP22BJ1 and RREP22BJ1)	This work
pABW1-REP22BJ2	pABW1 carrying REP the module of pA22BJ2 (PCR- amplified with the primers LREP22BJ2 and RREP22BJ2)	This work
pABW1-REP29J1	pABW1 carrying the REP module of pA29J1 (PCR- amplified with the primers LREP29J1 and RREP29J1)	This work
pABW1-REP46H1	pABW1 carrying the REP module of pA46H1 (PCR- amplified with the primers LREP46H1 and RREP46H1)	This work
pABW1-REP46H2	pABW1 carrying the REP module of pA46H2 (PCR- amplified with the primers LREP46H2 and RREP46H2)	This work
pABW1-REP62H1	pABW1 carrying the REP module of pA62H1 (PCR- amplified with the primers LREP62H1 and RREP62H1)	This work
pABW1-REP62H2	pABW1 carrying the REP module of pA62H2 (PCR- amplified with the primers LREP62H2 and RREP62H2)	This work
pABW1-REP7BH1	pABW1 carrying the REP module of pA7BH1 (PCR- amplified with the primers LREP7BH1 and RREP7BH1)	This work
pABW1-REP54BH1	pABW1 carrying the REP module of pA54BH1 (PCR- amplified with the primers LREP54BH1 and RREP54BH1)	This work
pBBR1MCS-2	Km^r^; *ori* pBBR1; *lacZ*α; MCS	[27]
pBBR1-Pil6	pBBR1MCS-2 carrying the PIL module of pA6H3 (PCR- amplified with the primers LPIL and RPIL)	This work
pBBR1-Pil46	pBBR1MCS-2 carrying the PIL module of pA46H2 (PCR- amplified with the primers LPIL and RPIL)	This work
pBBR1-Pil62	pBBR1MCS-2 carrying the PIL module of pA62H1 (PCR- amplified with the primers LPIL and RPIL)	This work
pMAT1	Km^r^; *ori* pBBR1; *sacB*; entrapment plasmid	[28]
pRK2013	Km^r^; helper plasmid carrying genes for conjugal transfer of RK2	[29]

^1^ Primer sequences are listed in Table S1; PIL—pilus assembly; REP—replication.

**Table 2:** General features of the *Pseudomonas* spp. plasmids identified in this study.

9	GenBank Accession Number	Host Strain	Plasmid Size (bp)	GC Content (%)	No. of Genes	Genetic Modules ^1^
pA4J1	MK376337	ANT_J4, ANT_J5, ANT_J7, ANT_J29, ANT_J9B	10,609	53.77	15	MOB, REP, TA
pA6H1	MK376338	ANT_H6	2076	53.37	3	REP
pA6H2	MK376339	ANT_H6	5999	43.69	9	REP, TA
pA6H3	MK376340	ANT_H6	6925	54.69	12	PIL, REP, TA
pA7BH1	MK376341	ANT_H7B	10,451	53.19	12	MOB, PAR, REP, TA
pA7J1	MK376342	ANT_J7, ANT_J17, ANT_J25, ANT_J7B, ANT_J15B	9794	50.61	13	MOB, PAR, REP, TA
pA16J1	MK376343	ANT_J16, ANT_J16B	28,896	56.00	27	MOB, PAR, REP, RM, TA, TER
pA22BJ1	MK376344	ANT_J16B, ANT_J22B	2914	50.72	4	REP
pA22BJ2	MK376345	ANT_J16B, ANT_J22B	7521	53.56	12	MOB, REP, TA
pA29J1	MK376346	ANT_J29	3214	58.03	3	MOB, REP
pA46H1	MK376347	ANT_H46	5039	57.63	6	MOB, REP, TA
pA46H2	MK376348	ANT_H46	6602	54.18	11	PIL, REP, TA
pA54BH1	MK376349	ANT_H54B	11,401	52.36	14	CAP, HSP, MOB, PAR, REP, TA
pA62H1	MK376350	ANT_H62	6949	54.94	12	PIL, REP, TA
pA62H2	MK376351	ANT_H62	76,906	53.35	74	PAR, REP, SUG, TA, TE

^1^ CAP—capsule polysaccharide export protein; HSP—heat shock protein; MOB—mobilization to conjugal transfer; PAR—partitioning; PIL—pilus assembly; REP—replication; RM—restriction–modification; SUG—type I polysaccharide secretion; TA—toxin–antitoxin; TE—transposable element; TER—tellurium resistance.

**Table 3:** Features of complete transposable elements identified in the pA62H2 plasmid.

TE	Family (Group)	Location in pA62H2 (Coordinates)	TE Length (bp)	IR (bp) ^1^	DR Sequence
IS*Psp13*	IS*4* (IS*4*)	6917–8340	1424	17/18	5’-GCTCCAAGAAC-3’
IS*Psp14*	IS*3* (IS*3*)	42,584–43,820	1237	22/27	5’-AAT-3’
Tn*5501*	Tn*3*	50,967–56,596	5630	36/38	Not determined
IS*Psp15*	IS*256*	60,847–62,210	1364	23/27	5’-CTT-3’

^1^ the number of identical nucleotides/summarized length of IR. DR—direct repeat; IR—inverted repeat.

## Supplementary Materials

The following are available online at https://www.mdpi.com/2073-4425/10/11/850/s1.
