# Supplementary material for: Diversity and Horizontal Transfer of Antarctic Pseudomonas spp. Plasmids
Source: Genes (Basel). 2019 Oct 28;10(11):850. doi: 10.3390/genes10110850 (PMC6896180; doi:10.3390/genes10110850)
Supplement: Supplementary file 1 [file genes-10-00850-s001.pdf]

genes

MDPI

Supplementary materials

Diversity and horizontal transfer of Antarctic *Pseudomonas* spp. plasmids

Krzysztof Romaniuk [1], Michal Styczynski [1], Przemyslaw Decewicz [1], Oliwia Buraczewska [1], Witold Uhrynowski [1], Marco Fondi [2], Marcin Wolosiewicz [1], Magdalena Szuplewska [1] and Lukasz Dziewit [1,*]

[1] Department of Bacterial Genetics, Institute of Microbiology, Faculty of Biology, University of Warsaw, Miecznikowa 1, 02-096 Warsaw, Poland
[2] Department of Biology, University of Florence, via Madonna del Piano 6, Sesto Fiorentino, 50019 Florence, Italy
* Correspondence: ldziewit@biol.uw.edu.pl; Tel.: +48-225-541-406

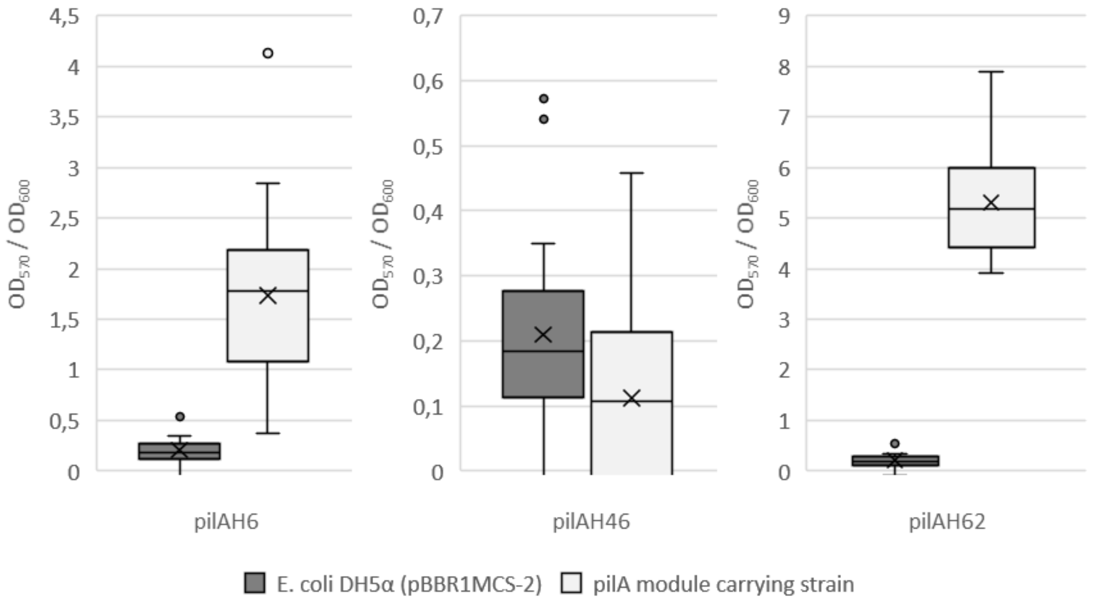

Figure S1. Box plots of OD_{570}/OD_{600} ratios for the results of the crystal violet staining test performed for *E. coli* DH5*α* (pBBR1MCS-2) and *E. coli* strains carrying PIL modules (*pilA* genes) of plasmids pA6H3, pA46H2 and pA62H1 cloned within the pBBR1MCS-2 vector (i.e. *E. coli* pilAH6, *E. coli* pilAH46 and *E. coli* pilAH62, respectively). The crosses correspond to the means. The central horizontal bars are the medians. The lower and upper limits of the box are the first and third quartiles, respectively. Points above and below the whiskers' upper and lower bounds are outliers. For the analysis the Mann-Whitney U test was applied.

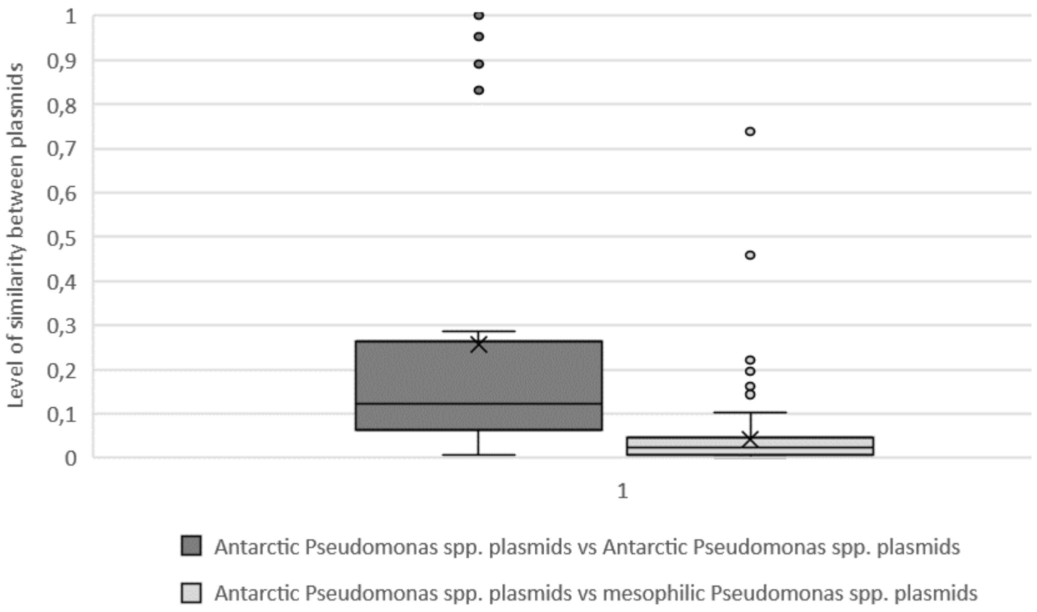

Figure S2. Box plots of levels of similarity between Antarctic *Pseudomonas* spp. plasmids and between Antarctic *Pseudomonas* spp. plasmids and mesophilic *Pseudomonas* spp. plasmids. The crosses correspond to the means. The central horizontal bars are the medians. The lower and upper limits of the box are the first and third quartiles, respectively. Points above and below the whiskers' upper and lower bounds are outliers. Statistical significance is of $p < 0.0001$. For the analysis the Mann-Whitney U test was applied.

genes MDPI

Table S1. Primers used in this study.

Primer	Sequence [1]	Plasmid	Position
A489SB	CAGACCGCTAACACAGTACA	pMAT1	3673-3692
A869SB	TTAGGATCTCCGGCTAATGC	pMAT1	4053-4034
AH62M95_F2	AGGTCGTGTGCCCAACGATG	pA62H2	55918-55899
AH62M95_F3	ACACCTTCGGTTGCGGGATG	pA62H2	55359-55340
AH62M95_F4	GATGTAAACGCGGCAACCTC	pA62H2	54777-54758
AH62M95_F5	AGGCGATCACTGGTGAGAAG	pA62H2	54181-54162
AH62M95_R2	ATGAGTTGCCGGTCGATCTG	pA62H2	51540-51559
AH62M95_R3	TACTGCTGGTAGGTACTCTC	pA62H2	52044-52063
AH62M95_R4	CCAACTCGTCGGACAAATGC	pA62H2	52507-52526
AH62M95_R5	TCGACCAGGTTGTCGGTGAG	pA62H2	53130-53149
AH62M95F	GTGTACGACTTGGCGTTGGG	pA62H2	56492-56473
AH62M95R	AAGGTGTAATGCCCGTTCAG	pA62H2	51060-51079
B1253SB	TTGTCGCCTGAGCTGTAGTT	pMAT1	4437-4418
B824SB	ACTATCACGGCTACCACATC	pMAT1	4008-4027
C1225SB	GATGAAGGCAACTACAGCTC	pMAT1	4409-4428
C1639SB	GACGATTGACGGCATTACGT	pMAT1	4823-4804
D1619SB	TGACGATTGACGGCATTACG	pMAT1	4803-4822
D1928SB	GACAGCATCCTTGAACAAGG	pMAT1	5112-5093
LPIL	tccagagctcGTTGCGAGAGGAAATACG	pA6H3	1615-1632
		pA46H2	1588-1605
		pA62H1	1616-1633
LRE22BJ2	atgaattcACCCAATCCACCGATAAG	pA22BJ2	7384-7401
LRE29J1	gcgaattcTGGCGCTGTTTAGGGTCTTG	pA29J1	2696-2715
LRE54BH1	gcgaattcGCAAGCCAAAGCGACGTGAC	pA54BH1	11325-11344
LREP16J1	ggcgaattcCGTAAGCGCAATCATACGAG	pA16J1	28841-28860
LREP22BJ1	gcgaattcTTCTGCGAGCATTGCCAAAG	pA22BJ1	2795-2814
LREP46H1	atgagctcTCGGTGGTGCTAAGTGATCC	pA46H1	4742-4761
LREP46H2	gcgaattcCCAGTGCTTCCCAAAGAG	pA46H2	6421-6438
LREP62H2	atggtaccCCTGTACGAGCGGATTCTTG	pA62H2	75494-75513
LREP6H1	cggaattcCAAGCTCGTACGGGTTCCAG	pA6H1	1977-1996
LREP6H2	ggcgaattcGATCCAGCTCGTTTGCATTG	pA6H2	5683-5702
LREP6H3	gcgaattcCGATCGCGCATTGAGAGTCG	pA6H3	6850-6869
		pA62H1	6874-6893
LREP7BH1	atggtaccGCTGCATCCGATCAATCAGG	pA7BH1	10218-10237
LREP7J1	gcgaattcAGGCAAAGCCGGTGACCATC	pA7J1	9643-9662
RPILE	tccaggatccGCTCGATCCACAAATTGC	pA6H3	4259-4242
		pA46H2	3935-3918
		pA62H1	4260-4243
RRE22BJ2	atggatccCGGGATTCAGGAAGAGAC	pA22BJ2	1610-1593
RRE29J1	gcaagcttTGGTTGAATCGCCGGAGATG	pA29J1	1358-1339
RRE54BH1	atggatccCCACCTGCACCCAGATCAAC	pA54BH1	2047-2028
RREP16J1	atggatccTGAGTGCTGCGCATGATGAG	pA16J1	1884-1865
RREP22BJ1	atggatccGCCTCAAGCGACGTATTTAG	pA22BJ1	1521-1502
RREP46H1	atggatccGACATGCCAGTCACGAATCC	pA46H1	1594-1575
RREP46H2	atggatccGATGTGTCGGGTCAAGTG	pA46H2	1523-1506

RREP62H2	atggatccTCTTGCGGACATCTGACGAC	pA62H2	1401-1382
RREP6H1	atggatccCCTCGATTACGGCGTCAGTG	pA6H1	1562-1543
RREP6H2	atggatccATAGTCTTCCAGCGCAGCAG	pA6H2	1378-1359
RREP6H3	atggatccGAGCGGTGCAAACCGTAGAG	pA6H3	1402-1383
		pA62H1	1401-1382
RREP7BH1	atggatccGGGCTGCTATCTTATCTAGG	pA7BH1	1462-1443
RREP7J1	atggatccAAATACGCTGGCCGTCTGGG	pA7J1	2012-1993

[1] Sequences are shown in the 5′ to 3′ orientation. Sequence not complementary to the plasmid are shown in lowercase. Introduced restriction sites are underlined.

Table S2. Summarization of the sequencing data for the *Pseudomonas* plasmids.

Plasmid name	GenBank accession no.	Contig no.	Number of reads	Covarage
pA4J1	MK379637	contig 1	619	14.2
pA6H1	MK379638	contig 1	1,485	172.6
pA6H2	MK379639	contig 1	819	36.6
pA6H3	MK379640	contig 1	903	33.7
pA7BH1	MK379641	contig 1	4,159	106.0
pA7J1	MK379642	contig 1	962	23.9
pA16J1	MK379643	contig 1	26,511	246.4
pA22BJ1	MK379644	contig 1	1,470	125.9
pA22BJ2	MK379645	contig 1	636	22.3
pA29J1	MK379646	contig 1	222	16.7
pA46H1	MK379647	contig 1	13,070	665.6
pA46H2	MK379648	contig 1	8,841	342.1
pA54BH1	MK379649	contig 1	2,839	66.9
pA62H1	MK379650	contig 1	7,699	282.1
pA62H2	MK379651	contig 1	1,592	142.7
		contig 2	23,813	169.4
		contig 3	190	43.2
		contig 4	1,210	52.3
		contig 5	590	51.9
		contig 6	699	65.4
		contig 7	740	50.5
		contig 8	790	67.0
		contig 9	130	27.2
		contig 10	220	40.1

genes

MDPI

Table S3. Genes located within the *Pseudomonas* spp. plasmids described in this study.

Gene name	Coding region (bp)	Orientation	Protein size (aa)	Possible function	Best BLAST hits		
					Percentage identity (aa)	Organism	GenBank accession no.
				**pA4J1**			
pA4J1_p...							
01	945-1,352	→	135	replication protein RepB	112/131 (81%)	*Pseudomonas fragi* (plasmid)	ARQ77166
02	1,621-1,379	←	80	hypothetical protein	72/79 (91%)	*Pseudomonas lundensis*	OZY34910
03	2,567-2,004	←	187	recombinase family protein	163/187 (87%)	*Pseudomonas fluorescens*	WP_106118790
04	3,364-2,801	←	187	transcriptional regulator, IscR family	165/186 (89%)	*Pseudomonas* sp. 286	WP_122782663
05	3,664-4,869	→	401	NAD(P)/FAD-dependent oxidoreductase	339/399 (85%)	*Pseudomonas fragi*	WP_095020670
06	5,579-5,313	←	88	toxin RelE	86/88 (98%)	*Pseudomonas versuta*	WP_073510837
07	5,790-5,563	←	75	antitoxin RelB	74/75 (99%)	*Pseudomonas versuta*	WP_073510839
08	6,283-6,651	→	122	DNA-binding protein	44/64 (69%)	*Pseudomonas* sp. 286	WP_122760497
09	7,444-7,175	←	89	hypothetical protein	39/79 (49%)	*Pseudomonas syringae*	WP_122232366
10	7,699-7,842	→	47	hypothetical protein, partial RepB	32/42 (76%)	*Pseudomonas* sp. LM12	AJW29839
11	8,178-7,960	←	72	hypothetical protein	35/72 (49%)	*Pseudomonas* sp. MYb115	WP_105755205
12	9,022-8,417	←	201	hypothetical protein	141/200 (71%)	*Pseudomonas lundensis*	OZY34828
13	10,170-9,085	←	361	mobilization protein MobA	308/357 (86%)	*Pseudomonas lundensis*	OZY34829
14	10,328-10,609	→	93	mobilization protein MobC	83/92 (90%)	*Pseudomonas weihenstephanensis*	WP_048403106
				**pA6H1**			
pA6H1_p...							
01	142-1,131	→	329	replication protein	177/214 (83%)	*Limnohabitans* sp. Rim47	WP_019431321
02	1,472-1,771	→	99	hypothetical protein	51/117 (44%)	*Acinetobacter baumannii*	WP_079768964
03	1,771-2,076	→	101	hypothetical protein	76/95 (80%)	*Pseudomonas aeruginosa*	KWZ63732

				**pA6H2**			
pA6H2_p...							
01	462-1,256	→	264	replication protein	144/278 (52%)	*Azotobacter beijerinckii*	WP_091014093
02	1,604-1,299	←	101	hypothetical protein	29/89 (33%)	*Acinetobacter baumannii*	WP_059264696
03	2,066-1,683	←	127	DNA-binding protein	87/145 (60%)	*Pseudomonas luteola*	WP_084339949
04	2,746-2,168	←	192	resolvase	149/188 (79%)	*Pseudomonas syringae*	WP_080034425
05	3,235-2,906	←	109	hypothetical protein	42/108 (39%)	*Enterobacter* sp. MGH85	KLW34121
06	3,312-4,133	←	273	hypothetical protein	77/79 (97%)	*Pseudomonas jessenii*	WP_110661030
07	4,441-4,154	←	95	toxin RelE	88/95 (93%)	*Pseudomonas mohnii*	WP_090470225
08	4,670-4,431	←	79	antitoxin RelB	77/79 (97%)	*Pseudomonas mohnii*	WP_090470222
09	5,795-4,752	←	347	hypothetical protein	213/304 (70%)	*Pseudomonas* sp. IB20	WP_094950509
				**pA6H3**			
pA6H3_p...							
01	560-1,267	→	235	replication initiator protein RepB	211/231 (91%)	*Pseudomonas lundensis*	OZY57286
02	1,350-1,757	→	135	hypothetical protein	109/138 (79%)	*Pseudomonas* sp. Eur1 9.41	WP_032489846
03	2,087-1,734	←	117	transcriptional regulator, XRE family	54/117 (46%)	*Pseudomonas savastanoi pv. glycinea*	RMQ98329
04	2,371-2,126	←	81	hypothetical protein	71/78 (91%)	*Pseudomonas lundensis*	OZY34910
05	2,658-2,368	←	96	hypothetical protein	71/95 (75%)	*Pseudomonas rhizosphaerae*	WP_043188100
06	3,262-2,825	←	145	major pilin protein PilA	87/139 (63%)	*Colwellia echini*	WP_101345566
07	3,922-3,452	←	156	hypothetical protein	82/150 (55%)	*Polaromonas* sp. CG9_12	WP_051983910
08	4,310-4,041	←	89	toxin RelE	86/89 (97%)	*Pseudomonas fluorescens*	AAG23808
09	4,518-4,294	←	74	antitoxin, TraY domain-containing	72/74 (97%)	*Pseudomonas fluorescens*	WP_024265244
10	4,784-5,200	→	138	antitoxin, HicB	105/138 (76%)	*Pseudomonas syringae*	SOS42876
11	5,814-6,050	→	78	hypothetical protein	38/75 (51%)	*Xenorhabdus bovienii*	WP_038196907
12	6,050-6,925	→	291	integrase	140/298 (47%)	*Pseudoalteromonas lipolytica*	WP_074989892
				**pA7BH1**			

pA7BH1_p...							
01	195-1,184	→	329	plasmid replication region	94/262 (36%)	*Bordetella avium*	WP_031943246
02	1,583-1,251	←	110	mobilization protein MobC	45/92 (49%)	*Pseudomonas aeruginosa*	WP_031631698
03	1,814-3,601	→	595	mobilization protein MobA	172/283 (61%)	*Pseudomonas weihenstephanensis*	WP_082149477
04	4,370-4,146	←	74	hypothetical protein	28/60 (47%)	*Pseudomonas*	WP_028700013
05	5,587-5,357	←	76	hypothetical protein	48/75 (64%)	*Pseudomonas stutzeri*	WP_015279002
06	6,377-5,814	←	187	resolvase	184/187 (98%)	*Pseudomonas* sp. ZM1	AIT41772
07	6,716-6,483	←	77	hypothetical protein	77/77 (100%)	*Pseudomonas stutzeri* RCH2	AGA88849
08	6,766-7,236	→	157	hypothetical protein	152/155 (98%)	*Pseudomonas* sp. ZM1	AIT41773
09	8,025-8,381	→	118	toxin RelE	84/119 (71%)	*Pseudomonas* sp. NFACC05-1	WP_092345609
10	8,374-8,691	→	105	antitoxin, transcriptional regulator YiaG	82/106 (77%)	*Pseudomonas* sp. NFACC05-1	WP_092345611
11	9,811-9,512	←	99	partitioning protein ParB	1155/1661 (70%)	*Azotobacter chroococcum*	ASL29126
12	10,449-9,811	←	212	partitioning protein ParB	55/88 (63%)	*Pseudomonas corrugata*	WP_055137501
				**pA7J1**			
pA7J1_p...							
01	164-910	→	248	replication initiator protein	248/248 (100%)	*Pseudomonas* sp. ANT_J3	AZQ19944
02	2,068-1,757	←	103	hypothetical protein	103/103 (100%)	*Pseudomonas* sp. ANT_J3	AZQ19945
03	2,352-2,065	←	95	mobilization protein MobC	95/95 (100%)	*Pseudomonas* sp. ANT_J3	AZQ19946
04	2,525-3,634	→	369	mobilization protein MobA	368/369 (99%)	*Pseudomonas* sp. ANT_J3	AZQ19947
05	3,679-4,272	→	197	mobilization protein MobB	197/197 (100%)	*Pseudomonas* sp. ANT_J3	AZQ19948
06	4,631-4,458	←	57	hypothetical protein	57/57 (100%)	*Pseudomonas* sp. ANT_J3	AZQ19949
07	5,657-5,253	←	134	hypothetical protein	134/134 (100%)	*Pseudomonas* sp. ANT_J3	AZQ19950
08	6,899-5,664	←	411	hypothetical protein	411/411 (100%)	*Pseudomonas* sp. ANT_J3	AZQ19938
09	7,273-7,887	→	204	resolvase	204/204 (100%)	*Pseudomonas* sp. ANT_J3	AZQ19939
10	8,235-7,891	←	114	toxin RelE	107/108 (99%)	*Pseudomonas* sp. ANT_J3	AZQ19940
11	8,476-8,195	←	93	antitoxin RelB	93/93 (100%)	*Pseudomonas* sp. ANT_J3	AZQ19941

12	8,879-9,523	→	214	partitioning protein ParA	214/214 (100%)	*Pseudomonas* sp. ANT_J3	AZQ19942
13	9,516-9,794	→	92	partitioning protein ParB	92/92 (100%)	*Pseudomonas* sp. ANT_J3	AZQ19943
				**pA16J1**			
pA16J1_p...							
01	279-1,025	→	248	replication initiator protein	232/247 (94%)	*Pseudomonas* sp. ANT_J3	AZQ19944
02	1,871-2,149	→	92	antitoxin ParD	84/91 (92%)	*Pseudomonas fragi*	WP_106116960
03	2,149-2,317	→	55	toxin RelE/ParE, partial	42/54 (78%)	*Pseudomonas syringae*	WP_017702649
04	4,707-2,569	←	712	methyl-accepting chemotaxis protein	412/439 (94%)	*Pseudomonas* sp. GV047	WP_108574867
05	5,209-5,400	→	63	antitoxin HiGA	38/62 (61%)	*Pseudomonas aeruginosa*	WP_057385177
06	5,725-5,438	←	95	hypothetical protein	60/91 (66%)	*Pseudomonas indoloxydans*	WP_108235078
07	6,210-5,776	←	144	mobilization protein MobC	124/137 (91%)	*Pseudomonas* sp. MF4836	WP_078474136
08	6,402-7,466	→	354	mobilization protein MobA/MobL	328/353 (93%)	*Pseudomonas* sp. MF4836	WP_078474137
09	7,493-8,137	→	214	mobilization protein MobB	198/215 (92%)	*Pseudomonas* sp. MF4836	WP_078474138
10	8,613-8,191	←	140	hypothetical protein	47/74 (64%)	*Pseudomonas* sp. ERMR1:02	PAM82820
11	10,778-9,867	←	303	hypothetical protein	90/313 (29%)	*Pseudomonas taeanensis*	WP_055725697
12	10,914-11,093	→	59	hypothetical protein	39/59 (66%)	*Pseudomonas kilonensis*	WP_116641939
13	14,364-11,242	←	1040	type I restriction endonuclease subunit R	1022/1040 (98%)	*Pseudomonas* sp. IB20	WP_094951733
14	15,413-14,361	←	350	restriction endonuclease-like (RecB) protein	337/350 (96%)	*Pseudomonas* sp. IB20	WP_094951734
15	16,741-15,410	←	443	type I restriction endonuclease subunit S	329/443 (74%)	*Candidatus Accumulibacter* sp. SK-01	KFB68175
16	18,464-16,731	←	577	type I restriction endonuclease subunit M	545/577 (94%)	*Pseudomonas syringae*	WP_099979039
17	18,668-19,051	→	127	hypothetical protein	83/130 (64%)	*Pseudomonas massiliensis*	WP_040259168
18	19,048-20,010	→	320	recombinase XerD	276/307 (90%)	*Pseudomonas veronii* 1YdBTEX2	SBW84942
19	20,162-20,380	→	72	hypothetical protein	42/72 (58%)	*Pseudomonas lundensis*	WP_082149930
20	20,970-20,665	←	101	toxin RelE/ParE, partial	51/54 (55%)	*Pseudomonas fragi*	WP_086799864
21	21,143-22,135	→	330	TerC family protein	319/330 (97%)	*Pseudomonas mandelii*	WP_010460781
22	24,906-22,456	←	816	hypothetical protein	634/813 (78%)	*Pseudomonas* sp. J237	OEO23875
23	26,444-24,903	←	513	NADH-quinone oxidoreductase subunit L	417/512 (81%)	*Pseudomonas marincola*	WP_090513455

24	26,548-27,432	→	294	transcriptional regulator, LysR family	268/289 (93%)	*Pseudomonas guineae*	WP_090242316
25	27,429-27,572	→	47	resolvase, partial	43/45 (96%)	*Pseudomonas* sp. MF4836	WP_078474142
26	27,980-28,624	→	214	partitioning protein ParA	204/214 (95%)	*Pseudomonas* sp. ANT_J3	AZQ19942
27	28,617-28,895	→	92	partitioning protein ParB	74/92 (80%)	*Pseudomonas* sp. ANT_J3	AZQ19943
				**pA22BJ1**			
pA22BJ1_p...							
01	315-1,367	→	350	replication protein	267/350 (76%)	*Pseudomonas* sp. S-47	WP_011266112
02	1,448-1,726	→	92	hypothetical protein	45/92 (49%)	*Pseudomonas aeruginosa*	WP_039026567
03	2,347-2,712	→	121	hypothetical protein	41/81 (51%)	*Pseudomonas anguilliseptica*	WP_090387993
04	2,666-2,896	→	76	hypothetical protein	31/66 (47%)	*Pseudomonas aeruginosa*	WP_034018119
				**pA22BJ2**			
pA22BJ2_p...							
01	945-1,352	→	135	replication protein RepB	112/139 (81%)	*Pseudomonas fragi*	ARQ77166
02	1,618-1,379	←	79	hypothetical protein	72/79 (91%)	*Pseudomonas fragi*	PAA00660
03	1,836-1,618	←	72	hypothetical protein	63/72 (88%)	*Pseudomonas frederiksbergensis*	WP_105339679
04	2,491-2,225	←	88	toxin RelE	86/88 (85%)	*Pseudomonas versuta*	WP_073510837
05	2,702-2,475	←	75	antitoxin RelB	74/75 (99%)	*Pseudomonas versuta*	WP_073510839
06	3,195-3,563	→	122	hypothetical DNA-binding protein	44/64 (69%)	*Pseudomonas* sp. 286	WP_122760497
07	4,356-4,087	←	89	hypothetical protein	39/79 (49%)	*Pseudomonas syringae*	WP_122232366
08	4,611-4,754	→	47	hypothetical protein, partial RepB	32/42 (76%)	*Pseudomonas* sp. LM12	AJW29839
09	5,090-4,872	←	72	hypothetical protein	35/72 (49%)	*Pseudomonas sp. MYb115*	WP_105755205
10	5,931-5,329	←	200	mobilization protein MobC	141/200 (71%)	*Pseudomonas lundensis*	WP_094990017
11	7,082-5,997	←	361	mobilization protein MobA/MobL	308/357 (86%)	*Pseudomonas lundensis*	WP_094990018
12	7,240-7,521	→	93	mobilization protein MobC	83/92 (90%)	*Pseudomonas weihenstephanensis*	WP_048403106
				**pA29J1**			
pA29J1_p...							

01	306-833	→	175	replication initiation protein	171/175 (98%)	*Pseudomonas fragi*	PAA29617
02	2,022-1,060	←	320	mobilization protein	310/320 (97%)	*Pseudomonas fragi*	WP_010657476
03	2,910-2,377	←	177	hypothetical protein	126/177 (71%)	*Pseudomonas reinekei*	WP_075945813
				**pA46H1**			
pA46H1_p...							
01	309-1,139	→	276	replication initiation protein RepB	232/270 (86%)	*Pseudomonas fragi*	PAA00661
02	1,407-2,078	→	223	hypothetical protein	113/141 (85%)	*Pseudomonas salomonii*	WP_069788611
03	2,153-2,353	→	66	antitoxin of TA system	66/66 (100%)	*Pseudomonas fragi*	PAA03537
04	2,340-2,606	→	88	toxin RelE	85/88 (97%)	*Pseudomonas fragi*	WP_095038416
05	4,396-2,603	←	597	mobilization protein MobA/MobL	291/458 (64%)	*Pseudomonas aeruginosa*	WP_077557595
06	4,538-4,888	→	116	mobilization protein MobC	83/114 (73%)	*Pseudomonas aeruginosa*	WP_077144139
				**pA46H2**			
pA46H2_p...							
01	536-1,240	→	234	replication initiator protein	211/231 (91%)	*Pseudomonas fragi*	PAA00381
02	1,322-1,729	→	135	hypothetical DNA-binding protein	118/137 (86%)	*Pseudomonas* sp. Eur1 9.41	WP_032489846
03	1,996-1,751	←	81	hypothetical protein	70/78 (90%)	*Pseudomonas fragi*	PAA00660
04	2,283-1,993	←	96	hypothetical protein	70/95 (74%)	*Pseudomonas rhizosphaerae*	WP_043188100
05	2,929-2,495	←	144	major pilin protein PilE	83/107 (78%)	*Aquincola tertiaricarbonis*	WP_046113134
06	3,596-3,123	←	157	hypothetical protein, sel1 repeat family	80/155 (52%)	*Variovorax paradoxus*	WP_080515766
07	3,985-3,716	←	89	toxin RelE	85/89 (96%)	*Pseudomonas fluorescens*	AAG23808
08	4,193-3,969	←	74	antitoxin, TraY domain-containing	71/74 (96%)	*Pseudomonas fluorescens*	WP_024265244
09	4,459-4,875	→	138	antitoxin HicB protein	106/138 (77%)	*Pseudomonas syringae*	SOS42876
10	5,467-5,727	→	86	hypothetical protein	38/75 (51%)	*Xenorhabdus bovienii*	WP_053460659
11	5,727-6,602	→	291	integrase	137/298 (46%)	*Pseudoalteromonas lipolytica*	WP_074989892
				**pA54BH1**			
pA54BH1_p...							

01	239-1,615	→	458	replication protein	361/456 (79%)	*Pseudomonas stutzeri*	WP_046622562
02	1,909-2,094	→	61	hypothetical protein	49/61 (80%)	*Pseudomonas stutzeri*	WP_046622535
03	2,379-2,104	←	91	hypothetical protein	78/91 (86%)	*Pseudomonas stutzeri*	WP_046622539
04	2,750-2,520	←	76	putative toxin	70/76 (92%)	*Pseudomonas stutzeri*	WP_015279002
05	2,925-2,734	←	63	antitoxin	57/63 (90%)	*Pseudomonas stutzeri*	WP_046622541
06	3,627-3,064	←	187	resolvase	181/187 (97%)	*Pseudomonas* sp. ZM1	AIT41772
07	3,966-3,733	←	77	hypothetical protein	77/77 (100%)	*Pseudomonas* sp. ARP3	WP_047882734
08	3,982-4,518	→	178	hypothetical protein	156/157 (99%)	*Pseudomonas* sp. ZM1	AIT41773
09	6,054-5,506	←	182	putative heat shock protein	167/182 (92%)	*Pseudomonas stutzeri*	WP_046622556
10	6,857-7,744	→	295	capsule polysaccharide export protein	234/295 (79%)	*Pseudomonas stutzeri*	WP_046622558
11	8,013-7,774	←	79	partitioning protein ParG	69/79 (87%)	*Pseudomonas stutzeri*	WP_046622560
12	8,606-8,016	←	196	partitioning protein ParA	188/196 (96%)	*Pseudomonas stutzeri*	WP_015279008
13	9,294-9,704	→	136	mobilization protein MobC	97/134 (72%)	*Pseudomonas stutzeri*	WP_015279007
14	9,701-11,362	→	553	mobilization protein, relaxase	414/559 (74%)	*Pseudomonas stutzeri*	WP_015279006
				**pA62H1**			
pA62H1_p...							
01	563-1,267	→	234	replication initiator protein RepB	211/231 (91%)	*Pseudomonas lundensis*	OZY57286
02	1,349-1,756	→	135	hypothetical protein	109/138 (79%)	*Pseudomonas* sp. Eur1 9.41	WP_032489846
03	2,086-1,733	←	117	transcriptional regulator, XRE family	54/117 (46%)	*Pseudomonas savastanoi pv. glycinea*	RMQ98329
04	2,370-2,125	←	81	hypothetical protein	71/78 (91%)	*Pseudomonas lundensis*	OZY34910
05	2,657-2,367	←	96	hypothetical protein	71/95 (75%)	*Pseudomonas rhizosphaerae*	WP_043188100
06	3,258-2,821	←	145	major pilin protein PilA	85/145 (59%)	*Alteromonadaceae bacterium* Bs31	WP_085153120
07	3,920-3,447	←	157	hypothetical protein	78/143 (55%)	*Variovorax paradoxus*	WP_080515766
08	4,309-4,040	←	89	toxin RelE	86/89 (97%)	*Pseudomonas fluorescens*	AAG23808
09	4,517-4,293	←	74	antitoxin, TraY domain-containing	72/74 (97%)	*Pseudomonas fluorescens*	WP_024265244
10	4,779-5,195	→	138	antitoxin, HicB	106/138 (77%)	*Pseudomonas syringae*	SOS42876
11	5,838-6,074	→	78	hypothetical protein	38/75 (51%)	*Xenorhabdus bovienii*	WP_038196907

12	6,074-6,949	→	291	integrase	139/298 (47%)	*Pseudoalteromonas lipolytica*	WP_074989892
				**pA62H2**			
pA62H2_p...							
01	305-1,051	→	248	replication initiator protein RepB	236/244 (97%)	*Pseudomonas* sp. 11/12A	WP_047539389
02	1,257-1,643	→	128	IS protein, AAA family ATPase, partial	103/113 (91%)	*Pseudomonas amygdali* pv. lachrymans	RMU22188
03	3,151-1,625	←	508	IS66 family transposase, Orf3	484/508 (95%)	*Pseudomonas syringae* group genomosp. 3	WP_122221446
04	3,550-3,215	←	111	IS66 family transposase, Orf2	107/111 (96%)	*Pseudomonas syringae*	WP_099264592
05	3,870-3,547	←	107	IS66 family transposase, Orf1	97/107 (91%)	*Pseudomonas* sp. 43NM1	WP_101209444
06	4,352-3,909	←	147	IS*3* family transposase, OrfB, partial	140/145 (97%)	*Pseudomonas* sp. GM78	EJN28358
07	6,139-4,790	←	449	IS*110* family transposase	427/449 (95%)	*Pseudomonas* sp. 25 R 14	WP_065942766
08	6,624-6,268	←	118	IS*3* family transposase, OrfA	106/118 (90%)	*Pseudomonas* sp. 25 R 14	CRM74011
09	6,977-8,332	→	451	IS*4* family transposase	417/451 (92%)	*Pseudomonas fluorescens*	KPU55444
10	8,371-8,586	→	71	hypothetical protein	47/57 (82%)	*Pseudomonas* sp. GM18	WP_007940411
11	9,811-8,690	←	373	glycosyltransferase	318/369 (86%)	*Pseudomonas* sp. 11/12A	WP_047539392
12	10,091-11,821	→	576	type I secretion system ABC permease	477/579 (82%)	*Pseudomonas moraviensis*	WP_024015088
13	11,829-13,160	→	443	type I secretion membrane fusion protein	348/443 (79%)	*Pseudomonas moraviensis*	WP_051600841
14	13,157-14,572	→	471	type I secretion outer membrane protein, TolC	391/469 (83%)	*Pseudomonas* sp. CFII64	WP_020294219
15	14,565-15,611	→	348	GDP-mannose 4,6-dehydratase	314/348 (90%)	*Paucimonas lemoignei*	WP_111758234
16	15,857-16,663	→	268	ABC-type polysaccharide phosphate export permease	171/268 (64%)	*Pseudomonas stutzeri*	WP_003292702
17	16,672-17,916	→	414	Teichoic acids export ATP-binding protein TagH	303/407 (74%)	*Pseudomonas stutzeri*	WP_031323302
18	18,177-20,684	→	835	glycosyltransferase, part 1	531/795 (67%)	*Pseudomonas stutzeri*	WP_024161821
19	20,681-21,157	→	158	glycosyltransferase, part 2	66/154 (42%)	*Pseudomonas stutzeri*	WP_024161821
20	21,159-22,253	→	364	class I SAM-dependent methyltransferase	180/364 (49%)	*Pseudomonas stutzeri*	WP_003293828
21	22,253-23,260	→	335	acyltransferase	203/329 (62%)	*Pseudomonas pseudoalcaligenes*	WP_104730062

22	23,733-23,503	←	76	hypothetical protein, partial IS630 transposase	29/51 (57%)	*Pseudomonas syringae*	WP_080501910
23	23,864-24,127	→	87	IS*66* family transposase, Orf1	58/65 (89%)	*Pseudomonas agarici*	SEL33180
24	24,144-24,467	→	107	group II intron reverse transcriptase/maturase, partial	104/107 (97%)	*Pseudomonas syringae pv. actinidiae*	RJX53357
25	24,501-24,704	→	67	IS*66* family transposase, Orf2	64/67 (96%)	*Pseudomonas* sp. 286	WP_122460747
26	24,719-24,868	→	49	IS*66* family transposase, partial	39/45 (87%)	*Pseudomonas syringae*	SFH67124
27	24,947-25,285	→	112	IS*66* family transposase, partial	106/112 (95%)	*Pseudomonas savastanoi*	WP_019742351
28	25,321-26,250	→	309	IS*66* family transposase, partial	260/278 (94%)	*Pseudomonas fragi*	WP_095024539
29	27,504-26,710	←	264	hypothetical protein	213/264 (81%)	*Pseudomonas veronii*	OPK04602
30	27,776-27,516	←	86	hypothetical protein	68/86 (79%)	*Pseudomonas veronii*	OPK04601
31	30,172-28,058	←	704	hypothetical protein	403/772 (52%)	*Diaphorobacter* sp. LR2014-1	WP_103738566
32	31,567-30,380	←	395	site-specific integrase/recombinase	326/395 (83%)	*Polaromonas* sp. OV174	WP_092004963
33	33,574-32,282	←	430	virulence-associated E family protein	235/402 (58%)	*Nitrosomonas ureae*	WP_074722219
34	34,897-34,466	←	143	hypothetical protein	59/143 (41%)	*Comamonadaceae bacterium* SCN 68-20	ODU56553
35	35,631-35,203	←	142	hypothetical protein	46/112 (41%)	*Sphingomonadales bacterium* EhC05	WP_066747373
36	38,456-35,826	←	876	calcium-binding protein	302/705 (43%)	*Aureimonas* sp. Leaf454	WP_056502841
37	39,975-39,013	←	320	GDP-mannose 4,6 dehydratase	250/315 (79%)	*Pseudomonas syringae*	WP_047580067
38	40,650-42,068	→	472	mannose-1-phosphate guanyltransferase	435/472 (92%)	*Pseudomonas syringae*	WP_047580063
39	43,402-42,593	←	269	IS*3* family transposase, OrfB	262/269 (97%)	*Pseudomonas plecoglossicida*	KGK23257
40	43,755-43,447	←	102	IS*3* family transposase, OrfA	100/102 (98%)	*Pseudomonas plecoglossicida*	KGK23256
41	43,822-46,359	→	845	diguanylate cyclase/phosphodiesterase	667/842 (79%)	*Pseudomonas* sp. CFII64	WP_020294342
42	46,576-46,782	→	68	hypothetical protein	53/67 (79%)	*Pseudomonas* sp. K1S02-6	WP_119956670
43	47,068-46,865	←	67	hypothetical protein	35/62 (56%)	*Pseudomonas frederiksbergensis*	WP_076030688
44	47,482-48,300	→	272	hypothetical protein	142/273 (52%)	*Pseudomonas* sp. Irchel 3E20	WP_095106811
45	48,423-48,854	→	143	hypothetical protein	90/143 (63%)	*Pseudomonas sp. HMSC08G10*	OFS75062
46	54,028-50,999	←	1009	Tn*3* family transposase	1008/1009 (99%)	*Klebsiella pneumoniae* MGH 39	EWF64779

47	54,614-54,012	←	200	Tn*3* resolvase	200/200 (100%)	*Pseudomonas putida* HB3267	AGA76234
48	54,806-55,183	→	125	toxin RelE	125/125 (100%)	*Pseudomonas putida* HB3267	AGA76233
49	55,164-55,499	→	111	antitoxin, XRE family transcriptional regulator	111/111 (100%)	*Pseudomonas putida* HB3267	AGA76232
50	55,514-55,849	→	111	hypothetical protein	111/111 (100%)	*Pseudomonas putida* HB3267	AGA76231
51	55,873-56,199	→	108	hypothetical protein	108/108 (100%)	*Pseudomonas putida* HB3267	AGA76230
52	56,302-56,556	→	84	hypothetical protein	84/84 (100%)	*Pseudomonas putida*	AHZ78114
53	56,597-56,887	→	96	hypothetical protein	96/96 (100%)	*Pseudomonas stutzeri* A1501	ABP80836
54	56,884-57,228	→	114	IS*66* family transposase, Orf1	114/114 (100%)	*Pseudomonas stutzeri* A1501	ABP80837
55	57,266-58,888	→	540	IS*66* family transposase, Orf3	540/540 (100%)	*Pseudomonas stutzeri*	KKJ93213
56	58,878-59,147	→	89	hypothetical protein	89/89 (100%)	*Pseudomonas stutzeri* A1501	ABP81004
57	59,252-59,653	→	133	hypothetical protein	133/133 (100%)	*Pseudomonas taiwanensis* SJ9	ESW37089
58	62,159-60,909	←	416	IS*256* family transposase	404/416 (97%)	*Pseudomonas fluorescens* F113	AEV60213
59	62,945-63,376	→	144	hypothetical protein	87/128 (68%)	*Pseudomonas* sp. 11BF10	ADU55740
60	63,548-63,793	→	81	hypothetical protein	54/73 (74%)	*Pseudomonas versuta*	WP_073514998
61	64,575-64,838	→	87	hypothetical protein	73/87 (84%)	*Pseudomonas frederiksbergensis*	SED93830
62	66,657-67,865	→	402	site-specific integrase/recombinase	337/397 (85%)	*Pseudomonas mohnii*	WP_090470228
63	68,862-67,837	←	341	replication terminus site-binding protein	248/343 (72%)	*Pseudomonas syringae*	WP_047579851
64	69,275-69,517	→	80	hypothetical protein	38/46 (83%)	*Stenotrophomonas rhizophila*	AXQ49145
65	70,451-69,918	←	177	class I SAM-dependent DNA methyltransferase	161/174 (93%)	*Pseudomonas aeruginosa*	WP_050413001
66	70,548-70,802	→	84	hypothetical protein	42/49 (86%)	*Pseudomonas* sp. A25(2017)	WP_077507182
67	71,147-70,881	←	88	hypothetical protein, truncated Rep	68/79 (86%)	*Pseudomonas putida*	ORL49904
68	71,774-71,463	←	103	hypothetical protein, truncated Rep	92/97 (95%)	*Pseudomonas putida*	WP_010895730
69	71,986-72,228	→	80	antitoxin PrlF	70/80 (88%)	*Pseudomonas* sp. VI4.1	OPK05999
70	72,225-72,620	→	131	toxin, PIN domain containing	112/130 (86%)	*Pseudomonas aeruginosa*	WP_033962410
71	73,143-73,012	←	43	hypothetical protein	40/43 (93%)	*Pseudomonas* sp. Irchel s3a12	WP_095126664

72	74,296-74,099	←	65	hypothetical protein	34/56 (61%)	*Klebsiella pneumoniae*	SVW35716
73	75,723-75,430	←	97	partitioning protein ParG	77/102 (75%)	*Pseudomonas mohnii*	WP_047539383
74	76,352-75,720	←	210	partitioning protein ParA	203/210 (97%)	*Pseudomonas* sp. 11/12A	WP_047539386

Table S4. Replication systems of Antarctic *Pseudomonas* plasmids identified in this study.

Plasmid name	*rep* gene (coordinates)	Sequences (and coordinates) of putative repeats of *oriV* site (DRs or IRs)[1]	Homologous REP protein (found based on the best BLASTP hit to the REP protein)
pA4J1	*pA4J1_01* (945-1,352)	IR1.1 – IR1.2: AAACCCTGCC(N_2)GGCAGGGTTT (698-719)<br>IR2.1 – IR2.2: CTGTAGGG(N_2)CCCTACAG (906-923)	*Pseudomonas fragi* (plasmid) [GenBank acc. no. APA32346]
pA6H1	*pA6H1_01* (142-1,131)	IR1.1 – IR1.2: 12-AAAGAGCCCCGAATCGACTCC(N_{22})GGGGTCGGTTCGGGGCTTCTTT (12-76)	*Limnohabitans* sp. Rim47 [GenBank acc. no. WP_019431321]
pA6H2	*pA6H2_01* (462-1,256)	DR1.1 – DR1.4: GCAATGTGCGAGA (322-334; 344-356; 366-378; 388-401)<br>DR2.1 – DR2.3: GATTCCTGT (335-343; 357-365; 379-387)	*Azotobacter beijerinckii* [GenBank acc. no. SFB60948]
pA6H3	*pA6H3_01* (560-1,267)	DR1.1 – DR1.4: GGGAGAGATTCCGGGT (392-407; 414-429; 436-451; 458-473)	*Pseudomonas lundensis* [GenBank acc. no. OZY57286]
pA7BH1	*pA7BH1_01* (195-1,184)	DR1.1 – DR1.2: ATTTGTTTGTTTA (98-111; 133-146)<br>DR2.1 – DR2.2: GTTTGTTTTGTT (119-130; 146-157)	*Bordetella avium* [GenBank acc. no. WP_031943246]
pA7J1	*pA7J1_01* (164-910)	DR1.1 – DR1.5: ACCCGGAATCYTTAGCTNCTT (897-918; 919-939; 940-961; 962-982; 983-1004)	*Pseudomonas* sp. ANT_J3 [GenBank acc. no. AQZ19944]
pA16J1	*pA16J1_01* (279-1,025)	DR1.1 – DR1.3: ACCCGGAATCCTTAGC (1012-1027; 1055-1070; 1098-1113)<br>DR2.1 – DR2.2: GCYTTAACCCGGAATCTTTAGCTACTT (1028-1054; 1071-1097)	*Pseudomonas* sp. ANT_J3 [GenBank acc. no. AQZ19944]
pA22BJ1	pA22BJ1_*01* (315-1,367)	IR1.1 – IR1.2: AAAAAGCCCCT(N_{30})AGGGGCTTTTT (34-73)<br>DR1.1 – DR1.2: TCACAGCGT (148-156; 158-166)	*Pseudomonas* sp. S-47 (plasmid) [GenBank acc. no. YP_232788]
pA22BJ2	pA22BJ2_*01* (945-1,352)	IR1.1 – IR1.2: AAACCCTGCCTTGGCAGGGTTT (698-719)<br>IR2.1 – IR2.2: CTGTAGGGCACCCTACAG (906-923)	*Pseudomonas fragi* (plasmid) [GenBank acc. no. APA32346]
pA29J1	*pA29J1_01* (306-833)	IR1.1 – IR1.2: GAAGCTCATAGCTGGTGAGCTTC (192-215)<br>IR2.1 – IR2.2: GAAGCTCACAGCTGGTGAGCTTC (218-241)	*Pseudomonas sp.* MF4836 [GenBank acc. no. OOV89046]
pA46H1	*pA46H1_01*	DR1.1 – DR1.4: GGGAGGGATTGACGGT (183-198 ;205-220; 227-242; 249-264)	*Pseudomonas lundensis*

	(309-1,139)	DR2.1 – DR2.3: TTAAAG (199-204; 221-226; 243-248)	[GenBank acc. no. OZY34914]
pA46H2	*pA46H2_01*	DR1.1 – DR1.5: GGGAGAGATTCCGGG (365-379; 387-401; 409-423; 431-445; 508-522)	*Pseudomonas lundensis*
	(536-1,240)	DR2.1 – DR2.3: TTAAAGG (380-386; 402-408; 424-430)	[GenBank acc. no. OZY57286]
pA54BH1	*pA54BH1_01*	DR1.1 – DR1.2: AGGGTCTGTCGCTACTACTAGG (1616-1637; 1663-1684)	*Pseudomonas stutzeri*
	(239-1,615)	DR2.1 – DR2.2: ATGCTTGC (1646-1653; 1796-1806)	[GenBank acc. no. WP_046622562]
pA62H1	*pA62H1_01*	DR1.1 – DR1.5: GGGAGAGATTCCGGG (392-406; 414-428; 436-450; 458-472; 535-549)	*Pseudomonas lundensis*
	(563-1,267)	DR2.1 – DR2.3: TTAAAGR (407-413; 429-435; 451-457)	[GenBank acc. no. OZY57286]
pA62H2	*pA62H2_01*	DR1.1 – DR1.4: TSTAWCGACTACAAATTCCGGCYATG (54-80; 82-108;176-202; 212-238)	*Pseudomonas sp.* 11/12A
	(305-1,051)		[GenBank acc. no. WP_047539389]

[1] Sequences are shown in the 5′ to 3′ orientation, DR – direct repeat, IR – inverted repeat, A – adenine, C – cytosine, G – guanine, T – thymine, Y – cytosine or thymine, R – adenine or guanine, W – adenine or thymine

genes

TABLE S5. Mobilization systems (including sequences of predicted origin of transfer – *oriT*) identified within the Antarctic *Pseudomonas* plasmids.

Plasmid name	MobA family	MOB genes (coordinates)	Sequences and coordinates of putative *oriT* site[1]
pA7J1	MOB_Q	*pA7J1_03-05* (2,065-4,272)	GCTGCAAACGTAGTTTGCGTAAGTGCGCCCTTCTC (2,429-2,466)
pA4J1	MOB_Q	*pA7J1_12-14* (8,417-10,609)	CCTGCAAACGTAGTTTGCGTAAGTGCGCCCTTCTT (10,214-10,248)
pA22BJ2	MOB_Q	*pA22BJ2_12-14* (5,329-7,521)	CCTGCAAACGTAGTTTGCGTAAGTGCGCCCTTCTT (7,126-7,160)
pA7BH1	MOB_Q	*pA7BH1_02-03* (1,251-3,601)	AGCAAGGGCGCACTTATGCAAACTTCGTTTGCGCG (1,746-1,712)
pA16J1	MOB_Q	*pA16J1_07-09* (5,776-8,137)	CGCCTTCTAAATTAGAAGGAAAAAGCGGACTATGT (6,267-6,301)
pA46H1	MOB_Q	*pA54BH1_05-06* (2,603-4,888)	CGTTTCTCGAAGAGAAACGCCATAGTGGCGCTCTC (4,396-4,429)
pA29J1	MOB_V	*pA29J1_01* (1,060-2,022)	CACTTCATAAGAAGTGTACTCACTAGACTTTGCTT (2,065-2,099)
pA54BH1	MOB_{HEN}	*pA54BH1_13-14* (9,294-11,362)	Not found

[1] Sequences shown in the 5′ to 3′ orientation.

TABLE S6. Partitioning systems (including sequences of predicted partitioning sites – *parS*) identified within the analyzed *Pseudomonas* plasmids.

Plasmid name	*par* genes (coordinates)	Sequences and coordinates of putative direct repeats (DR) of *parS* site [1]	Homologous PAR system (found based on the best BLASTP hit to the ParA protein)
pA7J1	*pA7J1_12-13* (8,879-9,794)	DR1.1 – DR1.8: ATTMDS (8,827-8,832; 8,833-8,838; 8,839-8,844; 8,845-8,850; 8,851-8,856; 8,857-8,862; 8,863-8,868; 8,869-8,874)	*Pseudomonas* sp. ANT_J3 (plasmid pA3J1) [GenBank acc. nos.: AZQ19942 and AZQ19942]
pA7BH1	*pA7BH1_11-12* (9,512-10,449)	DR1.1 – DR1.6: TRMRCA (9,439-9,444; 9,452-9,457; 9,461-9,466; 9,480-9,485; 9,489-9,494; 9,498-9,503)	*Pseudomonas flueorescens* R124 (plasmid pMP-R124) [GenBank acc. nos.: YP_008578813 and YP_008578814]
pA16J1	*pA16J1_26-27* (27,980-28,895)	DR1.1 – DR1.5: AATTM (27,944-27,948; 27,955-27,959; 27,962-27,966; 27,969-27,973; 27,976-27,980)	*Pseudomonas* sp. ANT_J3 (plasmid pA3J1) [GenBank acc. nos.: AZQ19942 and AZQ19942]
pA54BH1	*pA54BH1_11-12* (7,774-8,606)	DR1.1 – DR1.4: ACTCAT (8,597-8,602; 8,603-8,608; 8,609-8,614; 8,617-8,622)	*Pseudomonas stutzeri* RCH2 (plasmid pPSEST01) [GenBank acc. nos.: AGA88858 and AGA88859]
pA62H2	*pA62H2_73-74* (75,430-76,352)	DR1.1 – DR1.6: GCTA (75,406-75,409; 75,410-75,413; 75,414-75,417; 75,418-75,421; 75,422-75,425; 75,426-75,429)	*Pseudomonas* sp. 11/12A [GenBank acc. nos.: WP_047539384 and WP_047539386]

[1] Sequences are shown in the 5′ to 3′ orientation, DR – direct repeat, A – adenine, C – cytosine, G – guanine, T – thymine, M – adenine or cytosine, S – cytosine or guanine, R – adenine or cytosine, Y – cytosine or thymine, D – adenine, guanine or thymine.

TABLE S7. *Pseudomonas* plasmids used for the construction of the protein-based similarity network.

Id	Node label	Degree value	GenBank acc. no	Psychrotolerant	Organism (*Pseudomonas*)	Strain	Plasmid name	Plasmid size (bp)
n1	1	136	AB088420.3	no	*P. resinovorans*		pCAR1	199,035
n2	2	48	AB237655.1	no	*P. putida*	G7	NAH7	82,232
n3	3	116	AB238971.1	no	*P. putida*	MT53	pWW53	107,929
n4	4	78	AB434906.1	no	*P. putida*	HS1	pDK1	128,921
n5	5	136	AB474758.1	no	*P. putida*	CA10	pCAR1.2	200,231
n6	6	0	AB714582.1	no	*Pseudomonas* sp.	K-62	pMR68	71,020
n7	7	122	AE016854.1	no	*P. syringae* pv. tomato	DC3000	pDC3000B	67,473
n8	8	136	AE016855.1	no	*P. syringae* pv. tomato	DC3000	pDC3000A	73,661
n9	9	0	AF273219.1	no	*P. putida*		pYQ39	2,297
n10	10	124	AF359557.1	no	*P. syringae* pv. maculicola	M6	pFKN	39,554
n11	11	78	AF491307.2	no	*P. putida*	NCIB 9816-4	pDTG1	83,042
n12	12	0	AJ289784.1	no	*P. putida*	P8	pPP81	2,534
n13	13	100	AJ344068.1	no	*P. putida*		pWW0	116,580
n14	14	8	AJ421512.3	no	*Pseudomonas* sp.		pQBR55	5,924
n15	15	122	AJ877225.1	no	*P. aeruginosa*	Ps142	Rms149	57,121
n16	16	22	AM235768.1	no	*P. fluorescens*	SBW25	pQBR103	425,094
n17	17	102	AM261760.1	no	*P. aeruginosa*		pBS228	89,147
n18	18	54	AM778842.1	no	*P. aeruginosa*	07-406	pMATVIM-7	24,179
n19	19	140	AP013069.1	no	*P. resinovorans*	NBRC 106553	pCAR1.3	198,965
n20	20	0	AP014863.1	no	*P. furukawaii*	KF707	pKF707	59,815
n21	21	44	AP015030.1	no	*P. putida*	KF715	pKF715A	483,376
n22	22	112	AP015031.1	no	*P. putida*	KF715	pKF715B	276,165

n23	23	56	AP015032.1	no	*P. putida*	KF715	pKF715C	94,696
n24	24	6	AP015033.1	no	*P. putida*	KF715	pKF715D	30,071
n25	25	82	AY208917.2	no	*P. putida*	ND6	pND6-1	101,858
n26	26	6	AY257538.1	no	*P. aeruginosa*	C	pKLC102	103,532
n27	27	104	AY342395.1	no	*P. syringae* pv. syringae	A2	pPSR1	72,601
n28	28	122	AY603979.1	no	*P. syringae* pv. maculicola	ES4326	pPMA4326A	46,697
n29	29	122	AY603980.1	no	*P. syringae* pv. maculicola	ES4326	pPMA4326B	40,110
n30	30	0	AY603981.1	no	*P. syringae* pv. maculicola	ES4326	pPMA4326D	4,833
n31	31	4	AY603982.1	no	*P. syringae* pv. maculicola	ES4326	pPMA4326C	8,244
n32	32	0	AY603983.1	no	*P. syringae* pv. maculicola	ES4326	pPMA4326E	4,217
n33	33	78	AY887963.3	no	*P. fluorescens*	PC20	pNAH20	83,042
n34	34	0	AY951984.1	no	*Pseudomonas* sp.		p47L	3,084
n35	35	0	AY951985.1	no	*Pseudomonas* sp.		p47S	1,782
n36	36	2	CM001562.1	no	*P. fluorescens*	R124	pMP-R124	43,794
n37	37	100	CM001987.1	no	*P. syringae* pv. syringae	SM	pSM1	46,560
n38	38	128	CM002331.1	no	*P. moraviensis*	R28-S	pR28	81,846
n39	39	124	CM002754.1	no	*P. syringae* pv. actinidiae	ICMP 9617	unnamed	30,848
n40	40	16	CM003767.1	no	*P. aeruginosa*	BH6	pBH6	3,652
n41	41	10	CM007350.1	no	*P. aeruginosa*	PA3448	pPA3448	49,094
n42	42	14	CP003042.1	no	*P. fluorescens*	A506	pA506	56,977
n43	43	4	CP003072.1	no	*P. stutzeri*	RCH2	pPSEST01	12,763
n44	44	0	CP003073.1	no	*P. stutzeri*	RCH2	pPSEST02	9,865
n45	45	0	CP003074.1	no	*P. stutzeri*	RCH2	pPSEST03	2,804
n46	46	8	CP003589.1	no	*P. putida*	ND6	pND6-2	117,003
n47	47	112	CP003739.1	no	*P. putida*	PC9	pPC9	80,360
n48	48	140	CP003962.1	no	*Pseudomonas* sp.	VLB120	pSTY	321,653
n49	49	140	CP005961.1	no	*P. mandelii*	JR-1	unnamed	410,512

n50	50	106	CP005971.1	no	*P. syringae*	UMAF0158	unnamed	63,004
n51	51	110	CP006257.1	no	*P. syringae* pv. syringae HS191	HS191	unnamed	52,548
n52	52	96	CP007015.1	yes	*P. syringae*	CC1557	pCC1557	53,629
n53	53	72	CP007510.1	yes	*P. stutzeri*	19SMN4	pLIB119	107,733
n54	54	158	CP009975.1	no	*P. putida*	S12	pTTS12	583,900
n55	55	40	CP010893.1	no	*Pseudomonas* sp.	MRSN12121	pMRVIM0812	36,379
n56	56	0	CP010894.1	no	*Pseudomonas* sp.	MRSN12121	unnamed	21,121
n57	57	0	CP011111.1	no	*P. chlororaphis*	PCL1606	unnamed	16,587
n58	58	84	CP011370.1	no	*P. aeruginosa*	S04 90	unnamed	159,187
n59	59	142	CP011973.1	no	*P. syringae* pv. actinidiae	ICMP 18884	unnamed	74,423
n60	60	142	CP012180.1	no	*P. syringae* pv. actinidiae	ICMP 18708	unnamed	74,432
n61	61	18	CP013125.1	no	*P. mendocina*	S5.2	pPME5	252,328
n62	62	60	CP014061.1	no	*P. monteilii*	FDAARGOS_171	unnamed	60,588
n63	63	10	CP015000.1	no	*P. aeruginosa*	PA7790	pPA7790	49,021
n64	64	34	CP015601.1	yes	*P. antarctica*	PAMC 27494	pP27494_1	135,475
n65	65	6	CP015602.1	yes	*P. antarctica*	PAMC 27494	pP27494_2	30,116
n66	66	4	CP015640.1	no	*P. fluorescens*	L228	unnamed	77,900
n67	67	18	CP015879.1	no	*P. citronellolis*	SJTE-3	pRBL16	370,338
n68	68	0	CP015993.1	no	*Pseudomonas* sp.	TCU-HL1	pTCUH	12,690
n69	69	104	CP016215.1	no	*P. aeruginosa*	PA121617	pBM413	423,017
n70	70	98	CP016446.1	no	*P. putida*	IEC33019	pIEC33019	52,710
n71	71	86	CP016850.1	no	*Pseudomonas* sp.	TMW 2.1634	pL21564-1	99,718
n72	72	58	CP016851.1	no	*Pseudomonas* sp.	TMW 2.1634	pL21634-2	56,859
n73	73	140	CP017008.1	no	*P. syringae* pv. actinidiae	NZ-45	pPsa20586	76,088
n74	74	142	CP017010.1	no	*P. syringae* pv. actinidiae	NZ-47	pPsa22180a	74,424
n75	75	8	CP017011.1	no	*P. syringae* pv. actinidiae	NZ-47	pPsa22180b	55,944
n76	76	128	CP017294.1	no	*P. aeruginosa*	PA83	unnamed1	398,087

n77	77	42	CP017887.1	yes	*P. frederiksbergensis*	ERDD5:01	unnamed1	371,069
n78	78	168	CP018203.1	no	*P. syringae* pv. actinidiae	ICMP 9853	p9853_A	34,963
n79	79	162	CP018204.1	no	*P. syringae* pv. actinidiae	ICMP 9853	p9853_B	32,947
n80	80	48	CP018320.1	no	*P. frederiksbergensis*	AS1	unnamed	81,841
n81	81	0	CP018759.1	yes	*P. psychrotolerans*	PRS08-11306	pPRS08-11306	114,250
n82	82	138	CP019731.1	no	*P. syringae* pv. actinidiae	CRAFRU 12.29	unnamed	74,460
n83	83	142	CP019733.1	no	*P. syringae* pv. actinidiae	CRAFRU 14.08	unnamed	74,460
n84	84	172	CP019872.1	no	*P. syringae* pv. tomato	B13-200	pB13-200A	125,801
n85	85	144	CP019873.1	no	*P. syringae* pv. tomato	B13-200	pB13-200B	93,873
n86	86	138	CP019874.1	no	*P. syringae* pv. tomato	B13-200	pB13-200C	82,965
n87	87	10	CP020561.2	no	*P. aeruginosa*	CR1	pCR1	46,804
n88	88	0	CP020602.1	no	*P. aeruginosa*	E6130952	pJHX613	36,454
n89	89	56	CP021133.1	no	*P. fragi*	NMC25	unnamed1	79,891
n90	90	62	CP021134.1	no	*P. fragi*	NMC25	unnamed2	54,359
n91	91	54	CP021135.1	no	*P. fragi*	NMC25	unnamed3	16,437
n92	92	114	CP022563.1	no	*P. monteilii*	B5	pSH5-1	130,536
n93	93	102	CP024713.1	no	*P. syringae* pv. actinidiae	MAFF212063	pMAFF212063-A	68,316
n94	94	168	CP024714.1	no	*P. syringae* pv. actinidiae	MAFF212063	pMAFF212063-B	68,156
n95	95	68	CP025052.1	no	*P. aeruginosa*	PB353	pPB353_1	59,923
n96	96	68	CP025054.1	no	*P. aeruginosa*	PB354	pPB354_1	59,923
n97	97	142	CP026333.1	no	*Pseudomonas* sp.	XWY-1	pXWY	394,537
n98	98	144	CP026557.1	no	*P. amygdali* pv. morsprunorum	R15244	p1_tig4	81,536
n99	99	178	CP026559.1	no	*P. amygdali* pv. morsprunorum	R15244	p2_tig3	168,854
n100	100	136	CP026560.1	no	*P. amygdali* pv. morsprunorum	R15244	p3_tig5	45,535
n101	101	130	CP026561.1	no	*P. amygdali* pv. morsprunorum	R15244	p4_tig6	40,810
n102	102	142	CP026563.1	no	*P. avellanae*	R2leaf	p1_tig4	97,840
n103	103	168	CP026564.1	no	*P. avellanae*	R2leaf	p2_tig5	102,862

n104	104	138	CP026565.1	no	*P. avellanae*	R2leaf	p3_tig6	69,519
n105	105	148	CP026566.1	no	*P. avellanae*	R2leaf	p4_tig8	42,783
n106	106	80	CP026567.1	no	*P. avellanae*	R2leaf	p5_tig9	20,491
n107	107	76	CP027167.1	no	*P. aeruginosa*	AR_0356	unnamed3	165,365
n108	108	64	CP027168.1	no	*P. aeruginosa*	AR_0356	unnamed1	57,053
n109	109	156	CP027170.1	no	*P. aeruginosa*	AR_0356	unnamed2	438,531
n110	110	76	CP027173.1	no	*P. aeruginosa*	AR_0353	unnamed1	41,559
n111	111	110	CP027175.1	no	*P. aeruginosa*	AR_0230	unnamed1	71,782
n112	112	42	CP027478.1	no	*P. koreensis*	P19E3	p1	467,568
n113	113	18	CP027479.1	no	*P. koreensis*	P19E3	p2	300,131
n114	114	96	CP027480.1	no	*P. koreensis*	P19E3	p3	283,378
n115	115	0	CP027481.1	no	*P. koreensis*	P19E3	p4	2,827
n116	116	76	CP029091.1	no	*P. aeruginosa*	AR441	unnamed1	165,365
n117	117	64	CP029092.1	no	*P. aeruginosa*	AR441	unnamed2	57,052
n118	118	156	CP029094.1	no	*P. aeruginosa*	AR441	unnamed3	438,529
n119	119	104	CP029096.1	no	*P. aeruginosa*	AR439	unnamed2	437,392
n120	120	0	CP029708.1	no	*P. aeruginosa*	K34-7	pK34-7-1	4,440
n121	121	2	CP029714.1	no	*P. aeruginosa*	BH9	pBH6	41,024
n122	122	8	CP030914.1	no	*P. aeruginosa*	Y89	pY89	85,842
n123	123	38	CP032256.1	no	*P. aeruginosa*	AR_0111	unnamed	129,422
n124	124	0	CP032615.1	no	*Pseudomonas* sp.	DY-1	p.1	26,350
n125	125	142	CP032632.1	no	*P. syringae* pv. actinidiae	Shaanxi_M228	pM228	72,748
n126	126	2	CP032678.1	no	*Pseudomonas* sp.	Leaf58	pBASL58	904,163
n127	127	142	CP032870.1	no	*P. syringae* pv. actinidiae	P155	pLKQG722	77,771
n128	128	0	CP033772.1	no	*P. aeruginosa*	FDAARGOS_532	unnamed1	1,249
n129	129	2	CP033773.1	no	*P. aeruginosa*	FDAARGOS_532	unnamed2	1,089
n130	130	68	CP033834.1	no	*P. aeruginosa*	FDAARGOS_570	unnamed	36,032

n131	131	130	CP034079.1	no	*P. syringae* pv. pisi	PP1	pPP1-1	62,150
n132	132	116	CP034080.1	no	*P. syringae* pv. pisi	PP1	pPP1-2	54,993
n133	133	20	CP034081.1	no	*P. syringae* pv. pisi	PP1	pPP1-3	39,003
n134	134	40	CP034355.1	no	*P. aeruginosa*	IMP-13	pPYO_TB	130,306
n135	135	42	CP034538.1	no	*P. poae*	CAP-2018	unnamed	151,061
n136	136	80	DQ126685.1	no	*Pseudomonas* sp.	CT14	pCT14	55,216
n137	137	0	EU410482.1	no	*P. aeruginosa*	E1	pMM1	2,140
n138	138	54	FJ948173.1	no	*P. putida*	W2	pW2	76,494
n139	139	116	HM560971.1	no	*P. aeruginosa*		pUM505	123,322
n140	140	82	HM626202.1	no	*P. putida*	DOT-T1E	pGRT1	133,451
n141	141	48	JN248563.1	yes	*Pseudomonas* sp.	MC1	KOPRI126573	81,814
n142	142	142	JQ418525.1	no	*P. syringae*	B76	pB76-81	76,232
n143	143	78	JQ418534.1	no	*P. syringae*	NCPPB880	pNCPPB880-40	41,724
n144	144	0	JQ418535.1	no	*P. syringae*	PDDCC3357	pPDDCC3357-6	3,249
n145	145	76	JQ418536.1	no	*P. syringae*	PT14	pPT14-32	34,526
n146	146	62	JX891462.1	yes	*P. migulae*	D2RT	pD2RT	129,894
n147	147	56	KC189475.1	no	*P. aeruginosa*	COL-1	pNOR-2000	21,880
n148	148	4	KC542381.1	yes	*Pseudomonas* sp.	GLE121	pGLE121P1	6,899
n149	149	0	KC542382.1	yes	*Pseudomonas* sp.	GLE121	pGLE121P2	8,330
n150	150	8	KC542383.1	yes	*Pseudomonas* sp.	GLE121	pGLE121P3	39,583
n151	151	138	KC543497.1	no	*P. aeruginosa*	PA96	pOZ176	500,839
n152	152	12	KC609322.1	no	*P. aeruginosa*	ST1006	pPA-2	7,995
n153	153	18	KC609323.1	no	*P. aeruginosa*	ST308	pCOL-1	31,529
n154	154	50	KF840720.1	no	*P. putida*	LD209	pLD209	38,403
n155	155	140	LT222313.1	no	*P. cerasi*		p58T1	101,345
n156	156	138	LT222314.1	no	*P. cerasi*		p58T2	70,584
n157	157	172	LT222315.1	no	*P. cerasi*		p58T3	143,614

n158	158	120	LT222316.1	no	*P. cerasi*		p58T4	64,870
n159	159	122	LT222317.1	no	*P. cerasi*		p58T5	61,794
n160	160	162	LT222318.1	no	*P. cerasi*		p58T6	11,314
n161	161	114	LT599585.1	no	*P. veronii*	1YdBTEX2	PVE_plasmid	373,858
n162	162	166	LT963392.1	no	*P. syringae* pv. cerasicola		PP1	118,774
n163	163	170	LT963393.1	no	*P. syringae* pv. cerasicola		PP2	86,328
n164	164	166	LT963394.1	no	*P. syringae* pv. cerasicola		PP3	111,777
n165	165	128	LT963396.1	no	*P. cerasi*		PP1	127,474
n166	166	170	LT963397.1	no	*P. cerasi*		PP2	144,075
n167	167	136	LT963398.1	no	*P. cerasi*		PP3	81,323
n168	168	138	LT963399.1	no	*P. cerasi*		PP4	70,589
n169	169	120	LT963400.1	no	*P. cerasi*		PP5	48,121
n170	170	176	LT963401.1	no	*P. cerasi*		PP6	18,360
n171	171	124	LT963403.1	no	*P. syringae* pv. avii		PP1	43,975
n172	172	154	LT963404.1	no	*P. syringae* pv. avii		PP2	109,843
n173	173	176	LT963405.1	no	*P. syringae* pv. avii		PP3	108,842
n174	174	170	LT963406.1	no	*P. syringae* pv. avii		PP4	77,492
n175	175	110	LT963407.1	no	*P. syringae* pv. avii		PP5	41,285
n176	176	164	LT963410.1	no	*P. syringae*		PP1	110,420
n177	177	148	LT963411.1	no	*P. syringae*		PP2	90,464
n178	178	154	LT963412.1	no	*P. syringae*		PP3	86,369
n179	179	132	LT963413.1	no	*P. syringae*		PP4	78,348
n180	180	154	LT969519.1	no	*P. aeruginosa*		RW109 plasmid 1	555,265
n181	181	80	LT969521.1	no	*P. aeruginosa*		RW109 plasmid 2	151,612
n182	182	140	LT985193.1	no	*P. syringae*	CFBP 2116	PP2	61,361
n183	183	146	LT985194.1	no	*P. syringae*	CFBP 2116	PP3	83,816
n184	184	144	LT985195.1	no	*P. syringae*	CFBP 2116	PP4	82,845

n185	185	128	NC_019265.1	no	*P. savastanoi*	NCPPB 3335	pPsv48A	78,357
n186	186	116	NC_019266.1	no	*P. savastanoi*	NCPPB 3335	pPsv48B	45,220
n187	187	176	NC_019292.1	no	*P. savastanoi*	NCPPB 3335	pPsv48C	42,103
n188	188	0	NZ_CM000959.1	no	*P. syringae* pv. lachrymans	M301315	pMPPla107	967,397
n189	189	6	NZ_CP018048.1	no	*P. aeruginosa*	DN1	unnamed1	317,349
n190	190	2	NZ_CP027176.1	no	*P. aeruginosa*	AR_0230	unnamed2	1,350
n191	191	0	NZ_CP029095.1	no	*P. aeruginosa*	AR439	unnamed1	1,129
n192	pA3J1	6	KY498026	yes	*Pseudomonas* sp.	ANT_J3	pA3J1	9,794
n193	pA4J1	2	MK376337	yes	*Pseudomonas* sp.	ANT_J4	pA4J1	10,609
n194	pA6H1	0	MK376338	yes	*Pseudomonas* sp.	ANT_H6	pA6H1	2,076
n195	pA6H2	0	MK376339	yes	*Pseudomonas* sp.	ANT_H6	pA6H2	5,999
n196	pA6H3	4	MK376340	yes	*Pseudomonas* sp.	ANT_H6	pA6H3	6,925
n197	pA7BH1	4	MK376341	yes	*Pseudomonas* sp.	ANT_H7B	pA7BH1	10,451
n198	pA7J1	6	MK376342	yes	*Pseudomonas* sp.	ANT_J7	pA7J1	9,794
n199	pA16J1	8	MK376343	yes	*Pseudomonas* sp.	ANT_J16	pA16J1	28,896
n200	pA22BJ1	0	MK376344	yes	*Pseudomonas* sp.	ANT_J22B	pA22BJ1	2,914
n201	pA22BJ2	2	MK376345	yes	*Pseudomonas* sp.	ANT_J22B	pA22BJ2	7,521
n202	pA29J1	0	MK376346	yes	*Pseudomonas* sp.	ANT_J29	pA29J1	3,214
n203	pA46H1	0	MK376347	yes	*Pseudomonas* sp.	ANT_H46	pA46H1	5,039
n204	pA46H2	4	MK376348	yes	*Pseudomonas* sp.	ANT_H46	pA46H2	6,602
n205	pA54BH1	4	MK376349	yes	*Pseudomonas* sp.	ANT_H54B	pA54BH1	11,401
n206	pA62H1	4	MK376350	yes	*Pseudomonas* sp.	ANT_H62	pA62H1	6,949
n207	pA62H2	44	MK376351	yes	*Pseudomonas* sp.	ANT_H62	pA62H2	76,906
n208	208	58	U88088.2	no	*P. alcaligenes*	NCIB 9867	pRA2	32,743
